# A saturated reaction in repressor synthesis creates a daytime dead zone in circadian clocks

**DOI:** 10.1371/journal.pcbi.1006787

**Published:** 2019-02-19

**Authors:** Koichiro Uriu, Hajime Tei

**Affiliations:** Graduate School of Natural Science and Technology, Kanazawa University, Kakuma-machi, Kanazawa, Japan; Ecole Normale Supérieure, FRANCE

## Abstract

Negative feedback loops (NFLs) for circadian clocks include light-responsive reactions that allow the clocks to shift their phase depending on the timing of light signals. Phase response curves (PRCs) for light signals in various organisms include a time interval called a dead zone where light signals cause no phase shift during daytime. Although the importance of the dead zone for robust light entrainment is known, how the dead zone arises from the biochemical reactions in an NFL underlying circadian gene expression rhythms remains unclear. In addition, the observation that the light-responsive reactions in the NFL vary between organisms raises the question as to whether the mechanism for dead zone formation is common or distinct between different organisms. Here we reveal by mathematical modeling that the saturation of a biochemical reaction in repressor synthesis in an NFL is a common mechanism of daytime dead zone generation. If light signals increase the degradation of a repressor protein, as in *Drosophila*, the saturation of repressor mRNA transcription nullifies the effect of light signals, generating a dead zone. In contrast, if light signals induce the transcription of repressor mRNA, as in mammals, the saturation of repressor translation can generate a dead zone by cancelling the influence of excess amount of mRNA induced by light signals. Each of these saturated reactions is located next to the light-responsive reaction in the NFL, suggesting a design principle for daytime dead zone generation.

## Author summary

Light-entrainable circadian clocks form behavioral and physiological rhythms in organisms. The light-entrainment properties of these clocks have been studied by measuring phase shifts caused by light pulses administered at different times. The phase response curves of various organisms include a time window called the dead zone where the phase of the clock does not respond to light pulses. However, the mechanism underlying the dead zone generation remains unclear. We show that the saturation of biochemical reactions in feedback loops for circadian oscillations generates a dead zone. The proposed mechanism is generic, as it functions in different models of the circadian clocks and biochemical oscillators. Our analysis indicates that light-entrainment properties are determined by biochemical reactions at the single-cell level.

## Introduction

Circadian clocks in various organisms are composed of cell autonomous gene expression rhythms with a nearly 24-hour period. Transcriptional-translational feedback loops (TTFL) in single cells drive such rhythmic gene expression [1–3]. One of the most important roles of circadian clocks is to entrain behavioral and physiological rhythms in organisms to the light-dark (LD) cycle on earth. A light signal shifts the phase of the clocks by affecting the biochemical reactions in the TTFLs that regulate circadian gene expression. Such phase responses of circadian clocks to light signals allow their entrainment to the LD cycle.

Most organisms maintain their behavioral rhythms under constant dark (DD) conditions, indicating that their circadian clocks set subjective day and night. Subjective day and night under DD conditions can be defined by referring to the rhythms in an LD cycle. The phase responses of the circadian clocks to light signals have been examined by exposing organisms to short light pulses under DD conditions. Phase shift as a function of the timing of light exposure characterizes the entrainment property of the circadian clocks and is referred to as the phase response curve (PRC) [4, 5]. Intriguingly, the PRCs of different organisms have several common features [4, 6]. First, a light pulse at subjective morning advances the phase of the clock. Second, a light pulse at subjective evening and night delays the phase. Third, a light pulse at subjective daytime hardly changes the phase. This time window during daytime, when the phase of the clock is insensitive to light pulses, is referred to as the dead zone.

Previous theoretical studies revealed the PRC shape that is optimal for robust light entrainment. This optimal PRC is similar to those observed in several organisms and, remarkably, includes a dead zone during daytime [6–9]. This is because the presence of a dead zone increases the robustness of the clock against external fluctuations, such as fluctuation of light intensity [6] and daylight length [9], by reducing the responsiveness of circadian clock systems to external signals. However, the mechanism whereby a dead zone is created during daytime while preserving phase responses during the night remains unclear.

Two possible mechanisms may underlie the creation of a dead zone during daytime. One possibility is the gating of light input to reduce its influence on circadian clock genes [10, 11]. Gating is an elaborated mechanism, as it must be clock-dependent to distinguish day from night. Molecules that may be responsible for such gating have been identified previously [12, 13]. Gating makes circadian clocks robust against external perturbation as described above by reducing input signals detrimental to clock gene expression [6, 9]. However, gating is ineffective against internal perturbation that arises inside the gate, such as noise in gene expression and physiological states in cells [7, 8]. The alternative and more beneficial mechanism is the use of biochemical reactions in the TTFL to directly decrease the responsiveness of the phase of the clocks to stimuli during daytime. A dead zone formed in this way can make the clocks resistant to both external and internal perturbation because of the unresponsiveness of the phase of the clocks [7, 8]. Hence, some organisms should evolve to create a dead zone by biochemical reactions in TTFLs. Here we propose a design principle for creating such dead zones by analyzing the circadian clock systems of different organisms.

**Fig 1:**
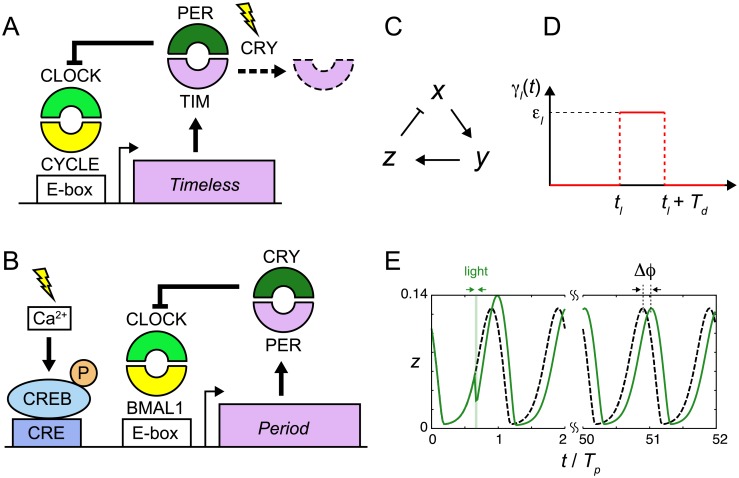
Modeling light responses of the circadian clocks. (A), (B) Schematic representation of the degradation and induction responses of the circadian clock. (A) Degradation response in *Drosophila* and (B) induction response in mammals. In (B), light signals increase Ca^2+^ levels. Subsequently, the elevation of Ca^2+^ increases the levels of phosphorylated CREB. See also the main text. (C) Negative feedback loop in the three-variable Goodwin model. *x*, *y* and *z* are the levels of repressor mRNA, cytoplasmic protein and nuclear protein, respectively. Nuclear protein represses mRNA transcription as indicated by a line with a perpendicular bar. (D) Change in a biochemical reaction by a light signal γ_*l*_(*t*) described by Eq (4). (E) Quantification of phase shift Δ*ϕ*. The green solid and black broken lines indicate time series of nuclear protein *z* with or without light perturbation, respectively. Time in the horizontal axis is normalized by the period of oscillation *T*_*p*_. We measure the difference in peak times after transient behaviors.

The biochemical reactions modulated by light signals in the TTFL for the circadian oscillation differ between species. In some organisms, such as *Drosophila*, light signals increase the degradation of circadian clock proteins, which we refer to as the degradation response (Fig 1A) [14, 15]. In *Drosophila*, the transcription of *Period* (*Per*) and *Timeless* (*Tim*) genes is induced by the CLOCK/CYCLE complex (Fig 1A). The PER/TIM complex then represses the transcriptional activity of CLOCK/CYCLE, forming a negative feedback loop (NFL) [16]. By this NFL, the abundance of TIM protein oscillates under both LD and DD conditions [14, 17]. Light signals activate *Chryptochrome* (*Cry*) and it degrades TIM protein [18–21]. This light-induced degradation of TIM allows the *Drosophila* clock to entrain to the LD cycle. As a result, the levels of TIM protein are lower during the day and higher during the night under LD conditions [14]. Differently, in other organisms such as mammals and *Neurospora*, light signals induce the transcription of repressor mRNA (Fig 1B) [10, 11, 22, 23]. In mammals, the transcription of *Per* and *Cry* is induced by the CLOCK/BMAL1 complex (Fig 1B). The PER/CRY complex then represses the transcriptional activity of CLOCK/BMAL1, forming an NFL as in the case of *Drosophila*. This NFL generates self-sustained rhythms of *Per* expression under both LD and DD conditions. Light signals induce the transcription of *Per* genes through the activation of CREB (Fig 1B) [10, 11, 24–26], allowing the mammalian clock to entrain to the LD cycle. In an LD cycle, *Per* expression levels are higher during the day and lower during the night [27]. We referred to this type of light response as an induction response.

Although light-responsive reactions differ between *Drosophila* and mammals, the mechanism for phase shifting at night is predicted to be the same: light signals induce repressor mRNAs when their concentrations are decreasing due to the strengthened feedback repression by the abundant repressor proteins. In *Drosophila*, degradation of TIM protein by light signals relieves transcriptional repression, leading to the induction of *Tim* mRNA. In mammals, light signals induce the transcription of *Per* via CREB. Thus, the elevation in repressor mRNA levels by light signals leads to phase shifts during the night in both systems.

On the other hand, there seems to be apparent differences in the effects of light signals on the dynamics of repressor mRNA and protein during daytime. In *Drosophila*, light signals increase the degradation of the repressor protein when its concentration is lower. In contrast, light signals in mammals increase repressor protein synthesis by inducing repressor mRNA when its concentration is already higher. These differences raise the question of whether the mechanisms for dead zone generation with different light responses are common or distinct. For the degradation response in *Drosophila*, several previous theoretical studies reproduced a dead zone without a clear explanation of its mechanism [28–30]. A reason for the elusiveness of this mechanism may be because an NFL with the degradation response can naturally create a dead zone without the inclusion of any additional reactions, as we discuss in this study. For the induction response in mammals, although various theoretical models have been proposed [29, 31–35], dead zone originated from unresponsiveness of phase of a clock has not been paid attention. Therefore, the mechanism underlying dead zone generation for the induction response observed in mammals should be clarified and compared with that for the degradation response observed in *Drosophila* to address the above questions.

Here, we reveal that the saturation of a biochemical reaction in the repressor synthesis in an NFL is a common mechanism to cancel the effect of light signals and create a dead zone in different organisms with the distinct light responses. The location of the saturated reaction in the NFL depends on the types of light responses. It is the saturation of repressor transcription that generates a dead zone with the degradation response, whereas it is the saturation of its translation with the induction response. In short, these saturated reactions in the repressor synthesis are located next to the light-responsive reactions in the NFL, suggesting a design principle for the dead zone generation during daytime.

## Methods

### Model for the degradation response

**Fig 2:**
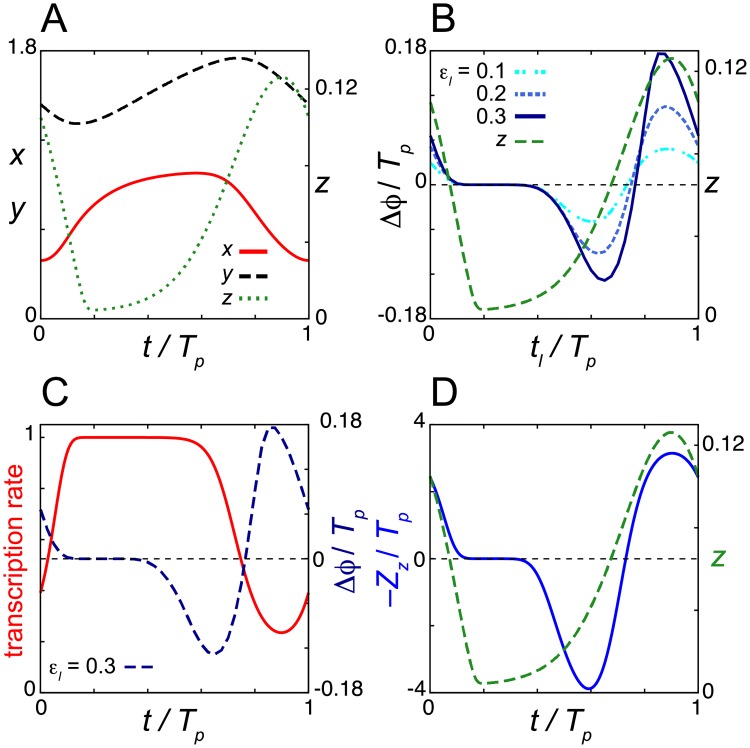
Dead zone generated by the saturation of repressor mRNA transcription in the degradation response. (A) Time series of the levels of mRNA *x*, cytoplasmic protein *y* and nuclear protein *z* in Eqs (1)–(3) without light signals. (B) Normalized phase response curves (PRC) to light signals. PRCs for different values of the rate of light-induced degradation *ε*_*l*_ are shown. (C) Time series of the transcription rate 1/(1+(*z*/*K*_1_)^*n*^) in Eq (1). The PRC for *ε*_*l*_ = 0.3 is also plotted (right y-axis) as a reference. (D) Phase sensitivity −*Z*_*z*_ (blue solid line). In (B) and (D), the time series of *z* (green broken line) is also plotted (right y-axis). In all panels, time is normalized by the period of oscillation *T*_*p*_ = 24. Values of reaction parameters in Eqs (1)–(3) are listed in S1 Table. In (B), *T*_*d*_ = 0.5*T*_*p*_/24 = 0.5.

We start with dead zone generation with the degradation response as observed in the *Drosophila* circadian clock (Fig 1A). As the neurons in the central pacemaker tissue are considered to determine the phase responses of individuals by entraining cells in peripheral tissues in general [36–39], we model a negative feedback loop in these pacemaker neurons. Because rhythms of these neurons are synchronized with each other by intercellular interactions [36, 40], they can be approximated as a single oscillator for simplicity. Previous theoretical studies reported a dead zone in PRCs with the degradation response [28–30]. However, which reaction processes are critical for the dead zone generation has not been clarified yet. To reveal the key determinants of the dead zone, we first consider the following dimensionless three-variable Goodwin model (Fig 1C):

$$\frac{1}{\tau }\frac{dx\left(t\right)}{dt}=\frac{1}{1+{\left(z\left(t\right)/K_{1}\right)}^{n}}-x\left(t\right),$$ (1)

$$\frac{1}{\tau }\frac{dy\left(t\right)}{dt}=\gamma_{1}x\left(t\right)-\gamma_{2}y\left(t\right),$$ (2)

$$\frac{1}{\tau }\frac{dz\left(t\right)}{dt}=\gamma_{2}y\left(t\right)-\left(\gamma_{3}+\gamma_{l}\left(t\right)\right)\frac{z\left(t\right)}{K_{m}+z\left(t\right)},$$ (3)

where *x*, *y* and *z* are the concentrations of the repressor mRNA, repressor protein in cytoplasm and repressor protein in nucleus, respectively. In this model, the repressor protein is translated in the cytoplasm and transported into the nucleus. In *Drosophila*, these variables correspond to the levels of *Tim* mRNA and proteins in each cell compartment. *K*_1_ and *n* in Eq (1) are the threshold and Hill coefficient for transcriptional repression, respectively. γ_1_ is the translation rate and γ_2_ is the transport rate of repressor protein from the cytoplasm to the nucleus. We assume the saturation of nuclear protein degradation in Eq (3). γ_3_ and *K*_*m*_ in Eq (3) are the maximum degradation rate of nuclear repressor protein and Michaelis constant, respectively. γ_*l*_ is the rate of degradation induced by transient light pulses and is specified below. The time constant *τ* can tune the period of oscillation without affecting other properties of a limit cycle. The three-variable model in the absence of light signals can generate stable limit cycles (Fig 2A). We set the origin of the horizontal axis in Fig 2 such that the levels of mRNA *x* take a minimum value at *t* = 0. The levels of *Tim* mRNA in *Drosophila* become lowest around dawn (~ CT 0) [17]. Hence, *t* = 0 in Fig 2 corresponds to the subjective dawn.

To examine the PRC, we consider the following form of light-induced perturbation in a reaction parameter (Fig 1D):

$$\gamma_{l}\left(t\right)=\{\begin{array}{cc} \varepsilon_{l} & t_{l}\leq t\leq t_{l}+T_{d}\\ 0 & \text{elsewhere} \end{array}$$ (4)

where *t*_*l*_ is the onset of a light pulse and *T*_*d*_ is the pulse duration. The parameter *ε*_*l*_ represents the rate of a light-induced biochemical reaction. For Eq (3), it is the rate of light-induced degradation of nuclear protein. We consider that *ε*_*l*_ reflects the strength of light. The value of *ε*_*l*_ becomes larger for a stronger light signal.

To obtain the light-induced phase shift Δ*ϕ*, we compute the difference in peak timing between perturbed and unperturbed systems (Fig 1E). A positive value of Δ*ϕ* indicates phase advance, whereas a negative value indicates phase delay. Typically, we run simulations for about 50 cycles after perturbation and measure the phase shift. Note that Δ*ϕ* quantifies the phase difference in terms of time. In this study, we examine the influence of each reaction parameter on the PRC. A change in the value of a reaction parameter may change the period of oscillation *T*_*p*_. For clearer comparison of PRCs between different parameter values, we compute the phase shift normalized by the period of oscillation, Δ*ϕ*/*T*_*p*_. In addition, the duration of the light pulse *T*_*d*_ in Eq (4) is also scaled with the period *T*_*p*_.

### Phase sensitivity

PRCs obtained by the above procedure may depend on the functional form of light-induced perturbation in biochemical reactions γ_*l*_(*t*). Therefore, it is desirable to characterize phase responses to perturbation based solely on a limit cycle of the unperturbed system as a complement. If a perturbation by a light signal is sufficiently small, the properties of a PRC can be well characterized by the phase sensitivity of a limit cycle [6, 41]. Phase sensitivity describes how a small increase in state variables at given time *t* shifts the phase of oscillation. Namely, the modulus of phase sensitivity represents the responsiveness of a clock to perturbation. Suppose *φ* is the phase of oscillation defined in radians (0 ≤ *φ* < 2 π) and **χ**(*φ*) = (*x*_*χ*_(*φ*), *y*_*χ*_(*φ*), *z*_*χ*_(*φ*)) is a limit cycle solution of Eqs (1)–(3) in the absence of perturbation. See S1 Text for the details of the definition of phase in the entire state space. A small perturbation to the state variables at time *t* can be described as **x**(*t*) = **χ**(*φ*(*t*)) + *μ* **η** where **η** is a unit vector that specifies the direction of perturbation in the state space and *μ* is the modulus of the perturbation (*μ* ≪ 1). Then, the phase shift δ*φ* caused by this perturbation reads:

$$\begin{array}{ll} \delta \varphi & =\varphi \left(\chi \left(\varphi \right)+\mu \eta \right)-\varphi \left(\chi \left(\varphi \right)\right)\\ & \approx {\mu \frac{\partial \varphi }{\partial x}|}_{x=\chi \left(\varphi \right)}\cdot \eta =\mu \tilde{Z}\left(\varphi \right)\cdot \eta , \end{array}$$ (5)

where $\tilde{Z}\left(\varphi \right)=\left({\tilde{\text{Z}}}_{x}\left(\varphi \right),{\tilde{\text{Z}}}_{y}\left(\varphi \right),{\tilde{\text{Z}}}_{z}\left(\varphi \right)\right)\equiv \partial \varphi \left(\chi \left(\varphi \right)\right)/\partial x$. Thus, the 2π periodic function $\tilde{Z}\left(\varphi \right)$ specifies the magnitude and direction of phase shift and is referred to as phase sensitivity [41]. A positive (negative) value of ${\tilde{Z}}_{i}$ (*i* ∈{*x*, *y*, *z*}) indicates that an infinitesimal increase of the variable *i* advances (delays) the phase of oscillation. Note that $\tilde{Z}\left(\varphi \right)$ can be determined for a limit cycle in the unperturbed system. With this phase sensitivity, the phase shift Δ*ϕ* quantified by a peak phase difference between perturbed and unperturbed systems can be approximated as (see S1 Text for details):

$$\Delta \phi \;\approx \frac{T_{p}}{2\pi }\int_{t_{l}}^{t_{l}+T_{d}}\tilde{Z}\left(\varphi \left(t\right)\right)\cdot G\left(t,\varphi \left(t\right)\right)\text{d}t,$$ (6)

where **G**(*t*, *φ*) is the perturbation in biochemical reactions by the light signal evaluated on the limit cycle **χ**. For example, **G**(*t*, *φ*) = (0, 0,–γ_*l*_(*t*)*z*_χ_(*φ*)/(*K*_*m*_+*z*_χ_(*φ*))) for Eqs (1)–(3). Hence, if ${\tilde{Z}}_{z}\left(\varphi \right)$ for Eqs (1)–(3) involves an interval where ${\tilde{Z}}_{z}\left(\varphi \right)\approx 0$, the interval will form a dead zone in the PRC. Although a dead zone can be formed in an interval where *G*_*z*_(*t*, *φ*) ≈ 0 as well, such dead zone formation was examined previously [9, 42] and is out of the scope of the present study. Because the phase shift Δ*ϕ* is measured as the peak time difference, we introduce $\text{Z}(\text{t})\;\equiv \;({\text{T}}_{\text{p}}/2\pi )\tilde{\text{Z}}(\varphi (\text{t}))$ to quantify the phase sensitivity in a unit of time. We compute the phase sensitivity for a limit cycle with the adjoint method as described in S1 Text.

## Results

### Dead zone with the degradation response

Fig 2B shows the PRC of the model Eqs (1)–(3) when a short light pulse is administered at each time point *t*_*l*_. We use pulse duration *T*_*d*_ = 0.5 *T*_*p*_/24 in Fig 2. For example, if *T*_*p*_ = 24h, *T*_*d*_ = 0.5h with this parametrization. During the night when the abundance of nuclear repressor protein *z* is higher, light signals shift the phase of oscillation (Fig 2B). A light pulse delays the phase of oscillation at which the levels of mRNA *x* are near their peak and those of nuclear repressor protein *z* are increasing (Fig 2B and S1A Fig). The reduction of repressor protein by a light signal during this time causes an increase in the transcription rate 1/(1+(*z*/*K*_1_)^*n*^), resulting in excess accumulation of mRNA (S1A Fig). Consequently, the nuclear repressor protein peaks later, delaying the initiation of the next cycle (S1A Fig). In contrast, a light pulse near the peak of *z* advances the phase of oscillation (Fig 2B and S1B Fig). The decrease in repressor protein during this time allows transcription to start earlier (S1B Fig). Thus, light signals induce the transcription of repressor mRNA by relieving transcriptional repression. The magnitude of phase shifts within these time windows becomes larger as the rate of light-induced degradation *ε*_*l*_ increases (Fig 2B). These results for the phase shifts are qualitatively consistent with previous experimental observations for the *Drosophila* circadian clock [14, 43].

In contrast, during daytime when the abundance of *z* is lower, the phase of oscillation does not change with light-induced degradation, indicating the presence of a dead zone (Fig 2B and S1C Fig). In this time window, the transcription rate of repressor is saturated at its maximum value due to the lower concentration of *z* (Fig 1C and S1C Fig). This saturation of transcription cancels the effect of light signals (S1C Fig), creating a dead zone. We also compute the phase sensitivity ***Z*** = (*Z*_*x*_, *Z*_*y*_, *Z*_*z*_). Because the nuclear protein *z* is decreased by a light signal through enhanced degradation, |*Z*_*z*_| is relevant to the magnitude of a phase shift. We consider –*Z*_*z*_ to match the phase advance and delay zones between phase sensitivity and the PRC (Fig 2D). –*Z*_*z*_ > 0 indicates phase advance by the decrease of *z* through light-induced degradation, while –*Z*_*z*_ < 0 indicates phase delay. The magnitude of *Z*_*z*_ is almost zero at the trough of nuclear protein concentration, confirming the existence of a dead zone (Fig 2D). The presence of a dead zone in phase sensitivity *Z*_*z*_ indicates that the dead zone in the PRC shown in Fig 2B is not created merely by the lower rate of light induced degradation ε_*l*_ *z*/(*K*_*m*_ + *z*) for *z* ≈ 0, but, indeed, lower phase sensitivity of the limit cycle to perturbation.

The continuous PRCs in Fig 2B are referred to as type 1. As the rate of light-induced degradation ε_*l*_ further increases, the PRC becomes discontinuous (S2A Fig), which is referred to as type 0. In S2A Fig, the breaking point (i.e., transition point of Δϕ/*T*_*p*_ from –0.5 to +0.5) is at around *t*_*l*_ / *T*_*p*_ = 0.85, where the levels of nuclear protein are near their peak. Even in this stronger light-induced degradation, a dead zone is maintained. The breaking point and the dead zone length are rather insensitive to the change in ε_*l*_ once the type 0 PRC is created (S2A Fig). With these larger values of ε_*l*_, the levels of repressor protein *z* become almost zero immediately after receiving a light pulse, meaning that the effect of light signals is saturated. In addition, the model can be entrained to a LD cycle (S2B Fig). The wave form of nuclear protein *z* peaks at night while reaching troughs in the daytime, which is consistent with experimental observations for TIM proteins under LD conditions [14, 15].

### Dependence of the dead zone length on reaction parameters of the degradation response

We then study how the dead zone length depends on the parameters in Eqs (1)–(3). We define the dead zone based on the magnitude of phase sensitivity **Z**. We detect a spanned time window where the phase of oscillation is insensitive to change in biochemical reactions induced by light signals:

$$\left\{t_{\text{1}}\leq t\;\leq t_{\text{2}}|\;\left|Z_{i}\left(t\right)\right|<\theta ,\left|Z_{i}\left(t_{\text{1}}\right)\right|=\left|Z_{i}\left(t_{\text{2}}\right)\right|=\theta \right\}\text{,}$$ (7)

where *i* ∈ {*x*, *y*, *z*} and *θ* is the threshold for phase irresponsiveness to perturbation. For the degradation response, phase sensitivity for nuclear protein *Z*_*z*_ is relevant to phase shifts by light signals, *i* = *z* in Eq (7). We set *θ* = 10^−1^ throughout the study. Any time window that satisfies Eq (7) is considered to be a dead zone and we measure its length *L*_*d*_ = (*t*_2_ –*t*_1_)/*T*_*p*_. Note that *L*_*d*_ is defined as the time interval normalized by the period of oscillation *T*_*p*_.

**Fig 3:**
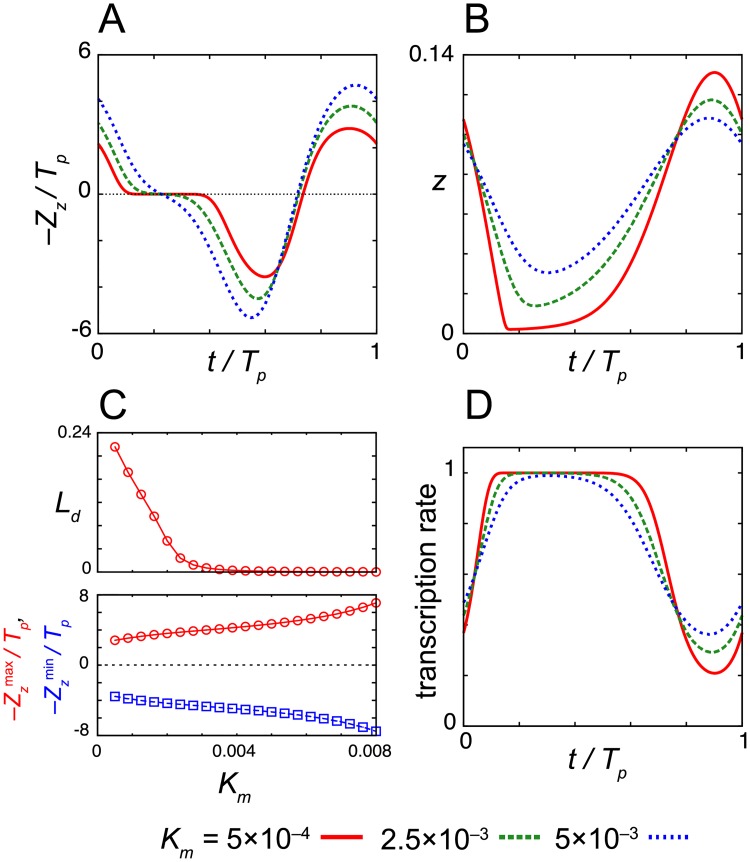
Dependence of the dead zone length on the Michaelis constant for nuclear protein degradation *K*_*m*_. (A) Phase sensitivity –*Z*_*z*_ and (B) time series of the levels of nuclear protein *z* for different values of *K*_*m*_. (C) Dead zone length *L*_*d*_ (top) and the maximum and minimum values of the phase sensitivity –*Z*_*z*_^(max)^ and –*Z*_*z*_^(min)^ (bottom) as a function of *K*_*m*_. In (A) and (C), the phase sensitivity is normalized by the period of oscillation *T*_*p*_. (D) Time series of transcription rate 1/(1+(*z*/*K*_1_)^*n*^) in Eq (1) for different values of *K*_*m*_. Values of reaction parameters are listed in S1 Table.

We first examine the dependence of the dead zone length *L*_*d*_ and the amplitude of phase sensitivity –*Z*_*z*_ on the Michaelis constant for protein degradation *K*_*m*_ (Fig 3). The amplitude of phase sensitivity decreases as the value of *K*_*m*_ decreases (Fig 3A and 3C). Instead, *L*_*d*_ monotonically increases as *K*_*m*_ decreases (Fig 3A and 3C). When the value of *K*_*m*_ is smaller and degradation is strongly saturated, the minimum levels of repressor proteins at troughs *z*_min_ are close to zero, *z*_min_/*K*_1_ ≪ 1 (Fig 3B). The effect of light signals diminishes at that time because the light-induced degradation of nuclear protein does not further increase the transcription rate, 1/(1+(*z*/*K*_1_)^*n*^) ≈ 1 in Eq (1) (Fig 3D, S1C and S3B Figs). Thus, the result confirms that the saturation of transcription is required for dead zone generation. The other requirement is quick recovery of the levels of nuclear protein after the light-induced degradation. If the recovery is slow, the duration of transcription is extended due to the lower levels of nuclear protein caused by light-induced degradation (S3A Fig). This longer duration of transcription results in phase shifts. These requirements are more likely to be satisfied when *z*_min_ ≈ 0. Thus, a dead zone tends to be long as the time interval where *z*(*t*) ≈ 0 becomes long. These results suggest that the strong saturation of TIM degradation lengthens the dead zone of the PRC in the *Drosophila* circadian clock.

Next, we study the dependence of the dead zone length *L*_*d*_ on the other parameters in Eqs (1)–(3) (S4 Fig; S1 Text). Typically, *L*_*d*_ depends on reaction parameters non-monotonically because the minimum value of nuclear protein *z*_min_ changes non-monotonically as the value of each parameter changes. In general, the amplitude of oscillation becomes smaller near a Hopf bifurcation point that sets the lower and upper bounds of an oscillatory parameter range. In the vicinity of Hopf bifurcation points, *z*_min_ is near the steady state and is more likely to be well above zero. Hence, *L*_*d*_ tends to be non-monotonic between the lower and upper Hopf bifurcation points. In addition, *z*_min_ becomes larger before the Hopf bifurcation points due to the accumulation of nuclear protein with, for example, faster nuclear protein transport (larger value of γ_2_) and slower degradation (smaller value of γ_3_), further reducing the dead zone length. The details of the dependence of *L*_*d*_ and amplitude of phase sensitivity –*Z*_*z*_ on each reaction parameter are described in S1 Text. For all the parameters, we find that *L*_*d*_ tends to be large when the values of *z*(*t*) at trough phase are close to zero. This observation suggests that each parameter influences the dead zone length by affecting the wave form of nuclear protein *z*(*t*).

In summary, the light-induced degradation of nuclear repressor protein induces transcription of the repressor mRNA. The elevation in mRNA levels result in phase shifts. A dead zone is formed if the light-induced degradation does not lead to a significant increase in *x*. Such time window arises when the nuclear protein concentration is significantly lower than the threshold for transcriptional repression *K*_1_. Thus, it is the saturation of repressor transcription that cancels the effect of light signals and creates a dead zone for the degradation response. Reaction parameters in the NFL affect the dead zone length by modulating the wave form of nuclear protein *z*.

### Dead zones in other oscillator models of the degradation response

To confirm the generality of the above results, we also analyze the dead zone in another model of *Drosophila* circadian clock [30]. The model includes interlocked feedback loops of PER/TIM and CLOCK/CYCLE. Qualitatively, the same results are obtained using this more complex *Drosophila* model (S5A–S5D Fig; S1 Text). Furthermore, we also consider a biochemical oscillator other than circadian clocks. We adopt a repressilator model for the synthetic oscillator [44]. We obtain same results using the repressilator model (S5E–S5H Fig; S1 Text), indicating that the proposed mechanism is robust and generic. Finally, we note that other previous models that realized dead zones also included the saturation of repressor mRNA synthesis and that of repressor degradation [28, 32, 45]. Thus, our current analysis highlights the relevance of saturation of repressor mRNA synthesis to dead zone formation.

### Model for the induction response

Next, we consider a model for the induction response (Fig 1B). As in the case of the degradation response, we model a negative feedback loop in central pacemaker neurons. In mammals, neurons in the suprachiasmatic nucleus (SCN) in the brain receive light signals from the eyes and determine the phase responses of individuals by entraining peripheral clocks [37–40]. Light signals induce the transcription of *Per* genes in these neurons. We describe this light response with the following dimensionless differential equations:

$$\frac{1}{\tau }\frac{dx\left(t\right)}{dt}=\frac{1}{\left(1+(z\left(t\right)/K_{1}{)}^{n}\right)}+\gamma_{l}\left(t\right)-x\left(t\right)$$ (8)

$$\frac{1}{\tau }\frac{dy\left(t\right)}{dt}=\gamma_{1}x\left(t\right)-\gamma_{2}y\left(t\right),$$ (9)

$$\frac{1}{\tau }\frac{dz\left(t\right)}{dt}=\gamma_{2}y\left(t\right)-\gamma_{3}\frac{z\left(t\right)}{K_{m}+z\left(t\right)},$$ (10)

where *γ*_*l*_(*t*) in Eq (8) is the induction rate of a clock gene by a light signal. *γ*_*l*_(*t*) is the same function as defined in Eq (4). The light signal induces transcription of repressor mRNA at rate *ε*_*l*_ independent of the concentration of repressor protein in Eqs (4) and (8). This may represent the induction of *Per* genes by CREB through CRE element in the mammalian circadian clock (Fig 1B) [24, 25]. The inclusion of light-induced transcription in this form differs from previous theoretical studies [6, 29, 32]. These previous studies assumed that the nuclear repressor protein also repressed the light-induced transcription. Therefore, in these models, the effect of light signals was diminished when the protein levels were high. Because light signals only influence the transcription of repressor mRNA in Eqs (8)–(10), phase sensitivity for *x*, *Z*_*x*_ underlies phase shifts.

**Fig 4:**
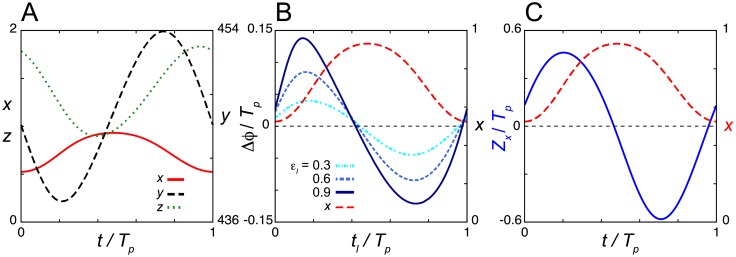
Phase response curves with no extended dead zone in the negative feedback loop model Eqs (8)–(10) with the induction response. (A) Time series of the levels of mRNA *x*, cytoplasmic protein *y* and nuclear protein *z* in Eqs (8)–(10) in the absence of a light signal. (B) Phase shift Δ*ϕ* as a function of the onset of light signals *t*_*l*_. Results for different values of *ε*_*l*_ are shown. (C) Phase sensitivity *Z*_*x*_. In (B) and (C), the time series of *x* (red broken line) is plotted (right y-axis) as a reference. Time is normalized by the period of oscillation *T*_*p*_. Values of reaction parameters are listed in S1 Table. *T*_*p*_ = 12.67. In (B), *T*_*d*_ = 0.5*T*_*p*_/24 = 0.3.

The model Eqs (8)–(10) generates stable limit cycles with appropriate parameter sets (Fig 4A). In Fig 4, we set *t* = 0 to the time at which the levels of mRNA *x* are at the minimum value. In the mammalian SCN, the expression levels of *Per* genes are lowest at around CT20 [27]. Hence, the origin of the horizontal axis in Fig 4 may correspond to the subjective midnight.

We then examine the phase shifts with the induction response (Fig 4B and 4C). We do not find an extended dead zone in either the PRC (Fig 4B) or the phase sensitivity *Z*_*x*_ (Fig 4C) of Eqs (8)–(10). Rather, Δ*ϕ* and *Z*_*x*_ intersect with zero steeply at a single time point. Phase delays are caused by light signals near the peak of mRNA. An increase in mRNA near its peak time results in an increase in the levels of nuclear protein and lengthens the duration of repression (S6 Fig). We further examine whether a dead zone is formed in Eqs (8)–(10) with other different parameter sets. For this, we randomly generate 2000 parameter sets from uniform distributions with which Eqs (8)–(10) can generate stable limit cycle oscillations (S1 Text). We compute the phase sensitivity *Z*_*x*_ for each random parameter set and check the length *L*_*d*_ of the spanned time window that satisfies the condition Eq (7). *L*_*d*_ of all the 2000 parameter sets examined is less than 1/24 (*e*.*g*., for *T*_*p*_ = 24h, *L*_*d*_ = 1/24 indicates a dead zone of 1h). Thus, our numerical results suggest that the NFL model Eqs (8)–(10) with the induction response does not form an extended dead zone in the PRC.

### Dead zone by saturation of repressor translation

**Fig 5:**
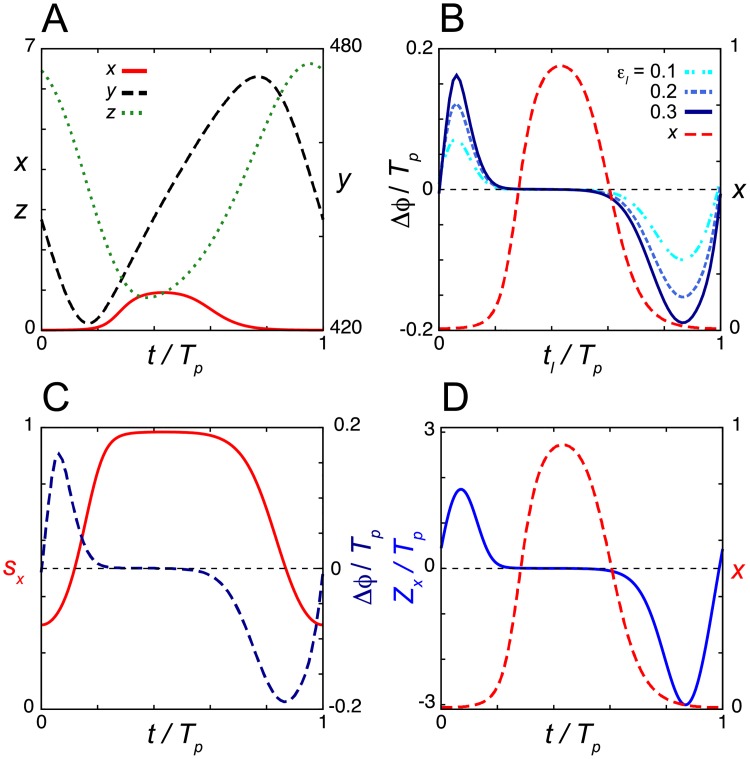
Dead zone generated by the saturation of repressor translation in the induction response. (A) Time series of the levels of mRNA *x*, cytoplasmic protein *y* and nuclear protein *z* in Eqs (8), (10) and (11) in the absence of a light signal. (B) Phase shift Δ*ϕ* as a function of the onset of light signals *t*_*l*_. Results for different values of *ε*_*l*_ are shown. (C) Time series of the saturation index *s*_*x*_ = *x*/(*K*_*t*_+*x*). The PRC with *ε*_*l*_ = 0.3 is also plotted (right y-axis). (D) Phase sensitivity *Z*_*x*_. In (B) and (D), the time series of *x* (red broken line) is plotted (right y-axis) as a reference. Values of reaction parameters are listed in S1 Table. *T*_*p*_ = 24. In (B), *T*_*d*_ = 0.5*T*_*p*_/24 = 0.5.

The analysis of the degradation response described in previous sections implies that a dead zone can be formed when a light signal does not increase the levels of nuclear repressor protein. For this, cancellation of the influence of mRNA induction by light signals may be required. This consideration leads us to introducing a saturation of a biochemical reaction in the NFL. We first test the saturation of protein transport from the cytoplasm to the nucleus, but it does not generate a dead zone (S7 Fig; S1 Text). We then test the saturation of mRNA degradation (S8 Fig; S1 Text). In this case, although a dead zone is formed at night when the concentration of repressor mRNA is lower, a daytime dead zone is not generated (S8 Fig). Finally, we introduce a Michaelis-Menten function in the translation process in Eq (9):

$$\frac{1}{\tau }\frac{dy\left(t\right)}{dt}=\gamma_{1}\frac{x\left(t\right)}{K_{t}+x\left(t\right)}-\gamma_{2}y\left(t\right),$$ (11)

where *K*_*t*_ is the Michaelis constant for translation. Translational regulation by certain RNA binding proteins [46, 47] may cause Michaelis-Menten type nonlinearity as assumed in Eq (11). Although a previous theoretical study examined the effect of a saturated translation term mainly on the period of oscillation [48], its effect on phase responses to light signals has not been studied. We simulate the model Eqs (8), (10) and (11), and find that it can generate sustained oscillations (Fig 5A). Remarkably, the saturated translation can generate an extended dead zone in the PRC at subjective daytime when the levels of mRNA *x*(*t*) are near their peaks (Fig 5B). The dead zone appears robustly even when we use different values of induction strength *ε*_*l*_ in Eqs (4) and (8) (Fig 5B). To quantify the degree of saturation, we define a saturation index for translation *s*_*x*_ = *x*(*t*)/(*K*_*t*_ + *x*(*t*)) [49]. The value of *s*_*x*_ is close to 1 when the translation is saturated and close to 0 when less saturated. The time series of *s*_*x*_ shows that the translation is indeed strongly saturated within the dead zone (Fig 5C). We also find that the dead zone is present in the phase sensitivity *Z*_*x*_ (Fig 5D). We then check whether other parameter sets can create similar dead zones. Of 2000 randomly generated parameter sets, 54 (2.7%) form similar dead zones of length *L*_*d*_ greater than 1/24. The reason for this relatively small percentage of longer dead zones is that the values of *K*_*t*_ are large in most of those 2000 random parameter sets, meaning that translation is not saturated strong enough to generate a longer dead zone. Larger values of *K*_*t*_ are favored because the saturation of translation tends to suppress oscillations [49], as we discuss in the discussion section.

Changes in time series of nuclear protein *z* generate phase shifts. The induction by light at the early increase phase of mRNA can increase the levels of nuclear protein near its trough (S9A Fig). Due to the excess amount of nuclear protein, the forthcoming peak of mRNA decreases. Accordingly, the levels of nuclear protein at the forthcoming peak are also decreased. Hence, the repression of mRNA synthesis relieves faster, advancing the phase. A light pulse at the late increase, the peak, and the early decrease phases of mRNA only weakly influences the levels of nuclear protein *z* due to saturation of translation (S9B Fig). An unaltered time series of *z* does not cause a phase shift. The induction of mRNA by light at the late decrease phase of mRNA can increase the peak levels of nuclear protein (S9C Fig). This lengthens the duration of transcriptional repression, delaying the phase.

This model can realize type 0 PRC at stronger light intensity (S10A Fig). Unlike the type 0 PRC in the degradation response (S2A Fig), the breaking point and shape of the PRC change depending on the strength of light induction *ε*_*l*_ (S10A Fig). Excess induction of mRNA lengthens the duration satisfying *x* > *K*_*t*_. This allows for the production of excess protein and delays its peaks, resulting in phase delays of greater magnitude (S10A Fig). We also confirm that the model can be entrained to an LD cycle (S10B Fig). The levels of mRNA peak during daytime in the LD cycle, which is consistent with experimental observations of *Per*s in mammals.

### Dependence of the dead zone length on parameters with the induction response

**Fig 6:**
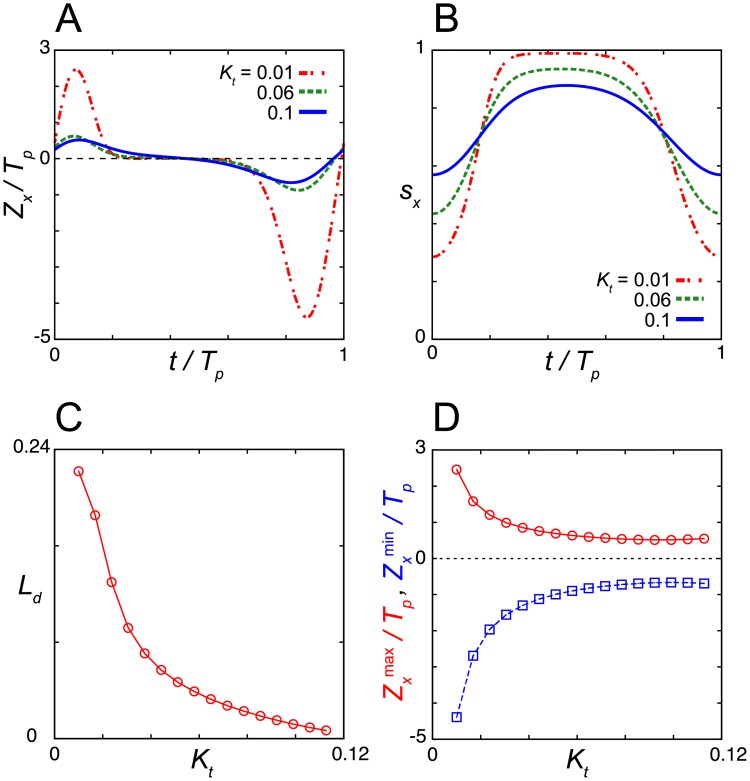
Dependence of dead zone length on the Michaelis constant for translation *K*_*t*_. (A) Phase sensitivity *Z*_*x*_ for different values of *K*_*t*_. (B) Time series of the saturation index *s*_*x*_ = *x*/(*K*_*t*_+*x*) for different values of *K*_*t*_. (C) Dead zone length *L*_*d*_ as a function of *K*_*t*_. (D) Dependence of the maximum and minimum values of the phase sensitivity *Z*_*x*_^(max)^ (red circles) and *Z*_*x*_^(min)^ (blue squares), respectively, on *K*_*t*_. *T*_*p*_ is the period of oscillation (*T*_*p*_ = 24). Values of reaction parameters are listed in S1 Table.

We then examine the parameter dependence of the dead zone length by computing the phase sensitivity *Z*_*x*_ for Eqs (8), (10) and (11) (Fig 6 and S11 Fig). We start with the dependence of *Z*_*x*_ on the Michaelis constant for translation *K*_*t*_ in Eq (11) (Fig 6A). Smaller values of *K*_*t*_ lead to stronger saturation of translation when the levels of mRNA are higher (Fig 6B). For each value of *K*_*t*_, we measure the length of a time window *L*_*d*_, within which the absolute value of phase sensitivity satisfies |*Z*_*x*_| < *θ* (*i* = *x* and *θ* = 10^−1^ in Eq (7)). *L*_*d*_ is larger for smaller values of *K*_*t*_ and it decreases monotonically with an increase in *K*_*t*_ (Fig 6C). The amplitude of *Z*_*x*_ also monotonically decreases as *K*_*t*_ increases (Fig 6D). Thus, the saturation of repressor translation increases both the dead zone length and phase sensitivity.

We next study the dependence of the dead zone length *L*_*d*_ and the amplitude of phase sensitivity *Z*_*x*_ on the other parameters in Eqs (8), (10) and (11) (S11 Fig). Overall, the dependence of *L*_*d*_ on each parameter is similar to that observed in the degradation response (Fig 3 and S4 Fig). The reason for this observation is as follows. In the induction response, the larger amplitude and wider wave form of repressor mRNA *x* extend the dead zone by lengthening the time interval during which translation is saturated. To achieve this condition, the levels of nuclear protein *z* at its trough must be near zero. This common requirement underlies the similarity in the parameter dependence of *L*_*d*_ between the degradation and induction responses.

Furthermore, as observed in the degradation response, the dead zone length *L*_*d*_ often changes non-monotonically as the value of a parameter increases (S11 Fig). For example, *L*_*d*_ depends on the maximum translation rate γ_1_ non-monotonically (S11A Fig). When γ_1_ is smaller, the amplitude of mRNA *x* is small, resulting in smaller *L*_*d*_ values. In contrast, when γ_1_ is larger, the amplitude of *x* is large whereas the width of its wave form narrows due to the higher levels of nuclear protein *z*. As a balance of these two contributions, *L*_*d*_ peaks near the lower Hopf bifurcation point (S11A Fig). A similar trend can be seen in the dependence on the transport rate γ_2_ where *L*_*d*_ peaks near the lower Hopf bifurcation point (S11B Fig) and the maximum degradation rate of nuclear protein γ_3_ where *L*_*d*_ peaks near the upper Hopf bifurcation point (S11C Fig). The dead zone length also depends non-monotonically on the threshold constant for transcriptional repression *K*_1_ (S11D Fig), as the amplitude of mRNA *x* changes non-monotonically between the lower and upper Hopf bifurcation points. As in the degradation response, the dead zone length becomes longer monotonically for smaller values of the Michaelis constant for protein degradation *K*_*m*_ (S11E Fig). The result indicates that the stronger saturation of protein degradation is more likely to lead to the generation of a dead zone.

Each reaction parameter also influences the amplitude of phase sensitivity *Z*_*x*_ (S11 Fig). Changes in the values of the maximum translation rate γ_1_ and protein degradation rate γ_3_ strongly influence the magnitude of phase delay rather than causing phase advancement (S11A and S11C Fig). Changes in the values of the other parameters affect the magnitudes of both phase advance and delay (S11B, S11D and S11E Fig). As observed in the degradation response, the amplitude of phase sensitivity becomes larger near the Hopf bifurcation points (S11 Fig).

### Hill function in translation

To further study the effect of nonlinearity in translation on the dead zone generation, we extend the Michaelis-Menten function in Eq (11) into a Hill function:

$$\frac{1}{\tau }\frac{dy\left(t\right)}{dt}=\gamma_{1}\frac{x{\left(t\right)}^{h}}{K_{t}{}^{h}+x{\left(t\right)}^{h}}-\gamma_{2}y\left(t\right),$$ (12)

where *h* is the Hill coefficient and the parameter *K*_*t*_ can now be interpreted as the threshold level of repressor mRNA required for translation to occur. Translation does not occur as long as *x*/*K*_*t*_ ≪ 1 for a large value of *h*. Time evolution of mRNA *x* and nuclear protein *z* are given by Eqs (8) and (10).

**Fig 7:**
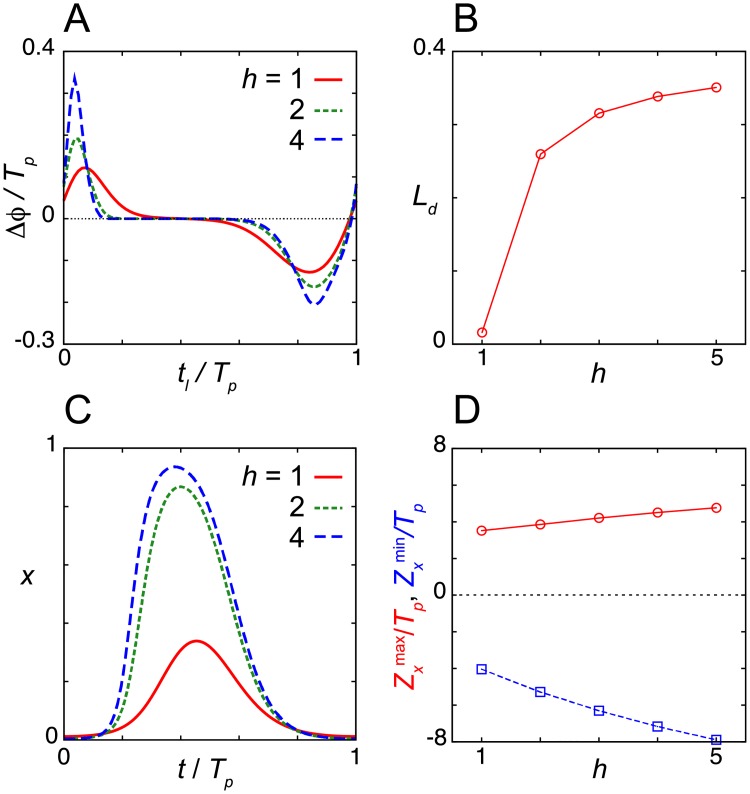
Dead zone generated by the Hill function for translation in the induction response. (A) Phase shift Δ*ϕ* as a function of the onset of light signals *t*_*l*_ for different values of the Hill coefficient *h* in Eq (12). (B) Dependence of the dead zone length *L*_*d*_ on *h*. (C) Time series of mRNA *x* for different values of the Hill coefficient *h*. (D) Dependence of the maximum and minimum values of phase sensitivity *Z*_*x*_^max^ and *Z*_*x*_^min^ on *h*. See S1 Table for values of reaction parameters. We set *T*_*p*_ = 24 by tuning *τ* for each value of *h*. In (A) *T*_*d*_ = 0.5*T*_*p*_/24 = 0.5 and *ε*_*l*_ = 0.1.

We study the dependence of the dead zone length on the Hill coefficient *h* with the same parameter set used in the analysis of the Michaelis-Menten translation function except for *K*_*m*_. For better illustration of the influence of *h*, we set *K*_*m*_ = 0.053 in Fig 7, which is larger than the value used in Fig 5 (*K*_*m*_ = 0.025). We find that the increase in *h* lengthens the dead zone (Fig 7A and 7B). This is because a larger *h* extends the interval of *x* where *x*^*h*^/(*K*_*t*_^*h*^+*x*^*h*^) ≈ 1. In addition, larger values of *h* increase the amplitude of *x* (Fig 7C). The amplitude of the PRC also increases with *h* (Fig 7A and 7D). The sharp transition of translational activity near *x* ~ *K*_*t*_ set by the Hill function more strongly influences the levels of nuclear protein *z*, resulting in a larger phase shift by a light signal. These results suggest that a switch in translation extends the dead zone in the PRC.

### Dead zones in other oscillator models of the induction response

Finally, to confirm the generality of the above results for the induction response, we analyze the dead zone in a more complex model of the mammalian circadian clock. The model includes the NFL of *Per* and *Bmal1* and that between *Bmal1* and *Rev-erb* (S1 Text). Saturation of translation creates a dead zone in this complex model (S12A and S12B Fig; S1 Text). In addition, we obtain a dead zone with the repressilator model including translational saturation and the induction response to external signals (S12C and S12D Fig; S1 Text). In summary, when light signals increase the mRNA synthesis independent of the clock states, the saturation of translation is required to generate the daytime dead zone.

## Discussion

The biochemical reactions that are influenced by light signals in circadian clock systems vary between organisms. In this study, we considered the degradation and induction responses as observed in *Drosophila* and mammals, respectively. Despite the difference in light responses in these two different animals, light signals induce the transcription of the repressor and cause phase shifts during night as described below. In the degradation response, light signals increase the degradation of repressor protein, thereby relieving transcriptional repression. Subsequent elevation of mRNA levels determines the phase shifts of the clock (S1 Fig). In the induction response where light signals directly induce the transcription of repressors, it is the subsequent increase in protein levels that determines the phase shifts (S6–S9 Figs). In contrast, a dead zone is formed in both types of light responses in the daytime. Previous studies demonstrated the importance of a dead zone during the daytime to make the circadian clock systems resistant against internal and external perturbation [6–9]. However, whether and how the TTFLs for the circadian clocks create the dead zone has remained unclear. In this study, we revealed that the saturation of a biochemical reaction in repressor synthesis is a common mechanism for the different light responses to reduce the phase sensitivity of a limit cycle and create a dead zone (Figs 2, 3 and 5). The degradation response requires the saturated transcription of repressor mRNA to generate a dead zone, whereas the induction response requires saturated repressor translation. Our theoretical results suggest that locating a saturated reaction in repressor synthesis next to a light-responsive reaction in an NFL is a design principle for dead zone generation.

The saturation of biochemical reactions in an NFL influences the generation of oscillations [49–51]. For example, the saturation of translation and transport of repressor protein from the cytoplasm to nucleus suppresses oscillations [49]. Conversely, the saturation of mRNA and protein degradation facilitates oscillations. Therefore, the location of the saturated reaction for dead zone generation should affect the ability of the NFL to generate oscillations. It is ideal if the saturated reaction for dead zone generation can facilitate generation of sustained oscillation. However, because the location of the saturated reaction for dead zone generation is constrained by that of the light-responsive reaction in an NFL as described above, it may not be optimal in terms of rhythm generation in some organisms. For the degradation response, the saturation of transcription at its maximum rate is required to create a dead zone (Figs 2C and 3D and S1C Fig). The saturated transcription can realize an effective transcriptional switch, as the resultant accumulation of repressor protein causes a subsequent rapid drop in the transcription rate (Fig 3D). Such a switch in transcription is favorable to oscillation, as the Hill function with a larger Hill coefficient in transcription facilitates oscillation [50, 52]. For the induction response, however, the saturation of translation is essential to create a dead zone (Figs 5 and 6). Although strong saturation of translation deprives the NFL of the ability to generate oscillation as described above (S13 Fig; S1 Text) [49], other additional saturations such as that of protein degradation could compensate (S13 Fig). Importantly, the saturation of protein degradation not only facilitates oscillations but also lengthens the dead zone in a PRC in both the degradation and induction responses (Fig 3, S3 and S11 Figs). A recent theoretical study proposed that the saturated degradation of molecules can be regarded as a positive feedback [51]. This suggests that there may be some positive feedbacks that support dead zone generation by the saturation of repressor synthesis, as the saturated degradation does.

Crucially, our analysis of the induction response indicates that not every saturated reaction in an NFL can create a dead zone during daytime. For example, the saturation of repressor transport from the cytoplasm to the nucleus does not generate a dead zone in the PRC (S7 Fig). The saturation of mRNA degradation does create a dead zone but only at night when the levels of mRNA are near the trough (S8 Fig). According to these results, the translation of repressor protein is the most plausible reaction to saturate for dead zone generation during the daytime (Fig 5). Additionally, we note that nonlinear functions of protein translation other than saturation cannot create a dead zone in the daytime, as we demonstrate that a Hill function for translation with a larger threshold *K*_*t*_ results in the generation of a dead zone at night (S14 Fig; S1 Text). A previous experimental study demonstrated that PER protein synthesis induced by vasoactive intestinal polypeptide (VIP) indeed saturated [53]. However, because the quantification was performed by the bioluminescence of the luciferase reporter fused with the functional PER2 protein, of which synthesis is under the control of endogenous transcriptional and translational regulations [54], it remains open whether this saturation occurred at the translation step of the PER protein. Recent experimental studies have revealed the importance of posttranscriptional regulations in the circadian clock [55]. Several micro RNAs inhibit the translation of clock gene transcripts by leading them to degradation. For example, micro RNAs regulate the onset of circadian rhythms in mouse embryonic tissue by determining the localization of *Clock* mRNA [56]. On the other hand, some protein molecules are known to promote translation. For example, mammalian LARK binds to *Per1* mRNA to promote translation of the PER1 protein [46]. Mouse heterogeneous nuclear ribonucleoprotein Q (mhnRNP Q) binds to 5'-UTR of *Per1* mRNA, which is necessary for internal ribosomal entry site mediated translation [47]. The saturated translation assumed in the present study may be caused by these mRNA binding proteins. Interestingly, although the levels of *Lark* and *mhnRNP Q* mRNAs are constant, the protein levels of both are rhythmic [46, 47]. Therefore, it is an important future work to reveal the significance of such rhythmic translational activities together with saturation in the phase responses of the circadian clock.

In general, a dead zone in the PRC can be generated in several ways. One way is to gate the light input to the circadian clock genes at a particular phase of oscillation. Because light signals do not influence the expression of circadian clock genes in the presence of gating, the phase sensitivity of the limit cycle does not need to be near zero to form a dead zone [9, 42]. Previous theoretical studies realized such gating by assuming that circadian clock proteins repress the light-induced transcription [9, 29, 42]. In mammals, light signals do not induce the expression of *Per* genes in the central pace maker tissue SCN during subjective day [10, 11], suggesting that the SCN gates the light input. However, the molecular mechanism of gating was not elucidated. Moreover, if the circadian system only utilizes gating, the system would remain susceptible to internal perturbation such as fluctuations in gene expression and physiological states in cells. In this study, we proposed an alternative mechanism of dead zone generation that is effective against both external and internal fluctuations [6–9] and functions in different organisms with different light responses. We note that the mechanism proposed in the present study can function together with gating and further improve clock precision. A dead zone like interval has been observed in the PRC of firing rate of neurons in the rat SCN explants treated with VIP for few minutes [57]. PRCs of single cells derived from peripheral tissues are typically type 0 [58–61], most likely due to strong phase resetting effects of applied perturbations (S10 Fig). A dead zone tends to be obscured in a type 0 PRC (S10 Fig), making it hard to perform direct comparison with type 1 PRCs of the current study. A future study will address whether SCN can create a dead zone at a single cell level by the saturated translation of *Per* mRNAs. Treatment of lower concentration of VIP or forskolin, which also activates CREB, to a cell line derived from the rat SCN [62] can be used to realize type 1 PRCs in single cells and address this question.

In conclusion, the saturation of a biochemical reaction in repressor synthesis is a simple and generic mechanism for the generation of a dead zone for light signals. Several environmental cues other than light signals change the phase of the circadian clock by influencing the rates of biochemical reactions. The PRCs for those cues may include a dead zone as observed in the responses to light pulses [63]. Our findings indicate that the saturation of biochemical reactions should also function in such dead zone generation.

## Supporting information

S1 Text Supplementary information. (PDF)

S1 Table Values of reaction parameters in the models. (PDF)

S1 Fig Phase shifts by light-induced degradation of nuclear repressor protein. (A)-(C) Time series of mRNA *x* and nuclear protein *z* (left) and that of transcription rate 1/(1+(*z*/*K*_1_)^*n*^) in Eq (1) in the main text (right) for the degradation response. Light is administered at (A) *t*/*T*_*p*_ = 0.65, (B) *t*/*T*_*p*_ = 0.85 and (C) *t*/*T*_*p*_ = 0.2 where *T*_*p*_ = 24 is the period of oscillation. Time series of *x*, *z* and transcription rate with a light signal (perturbed) and those in the absence of a light signal (unperturbed) are shown. Values of reaction parameters in Eqs (1)–(3) are the same as those in Fig 2 in the main text (S1 Table). *ε*_*l*_ = 0.3 and *T*_*d*_ = 0.5*T*_*p*_/24 = 0.5.
(TIFF)

S2 Fig Type 0 PRC and the entrainment of oscillation to an LD cycle with the degradation response. (A) Phase shifts Δ*ϕ* as a function of time *t*_*l*_ at which a light signal is administered. Results for different values of rate of light-induced degradation *ε*_*l*_ are plotted. Note that the results for *ε*_*l*_ = 3 and 6 are overlapped. Time series of nuclear protein *z* (green broken line) is also shown as a reference. *T*_*d*_ = 0.5*T*_*p*_/24 = 0.5. (B) Time series of mRNA *x*, nuclear protein *z* and light-induced degradation γ_*l*_ under a 12:12 LD cycle. White boxes indicate time windows where light is on and black boxes indicate those where light is off. The free running period is set to 24.3 with *τ* = 0.2818 in Eqs (1)–(3). Values of parameters in Eqs (1)–(3) are the same as those in Fig 2 (S1 Table).
(TIFF)

S3 Fig Phase shifts by light-induced degradation of nuclear repressor protein for different values of Michaelis constant *K*_*m*_. (A), (B) Time series of nuclear protein *z* and that of transcription rate 1/(1+(*z*/*K*_1_)^*n*^) in Eq (1) in the main text for the different values of Michaelis constant *K*_*m*_ for nuclear protein degradation. (A) *K*_*m*_ = 5×10^−3^ and (B) *K*_*m*_ = 5×10^−4^. Light is administered at *t*/*T*_*p*_ = 0.4 where *T*_*p*_ = 24 is the period of oscillation. Time series of *z* and transcription rate with a light signal (perturbed) and those in the absence of a light signal (unperturbed) are shown. Values of the other reaction parameters in Eqs (1)–(3) are the same as those in Fig 3 (S1 Table). *ε*_*l*_ = 0.6 and *T*_*d*_ = 0.5*T*_*p*_/24 = 0.5.
(TIFF)

S4 Fig Dependence of the dead zone length on each reaction parameter in the model with the degradation response Eqs (1)–(3). Dependence on (A) translation rate *γ*_1_, (B) protein transport rate *γ*_2_, (C) degradation rate of nuclear protein *γ*_3_ and (D) threshold constant for transcriptional repression *K*_1_. The first column shows the phase sensitivity –*Z*_*z*_ for different values of the focal parameter. The second column shows the time series of nuclear protein *z*. Line types correspond to those in the first column. The third column shows the dead zone length *L*_*d*_ as a function of the focal parameter. The fourth column shows minimum and maximum values of the phase sensitivity –*Z*_*z*_^min^ and –*Z*_*z*_^max^, respectively. The vertical dotted lines in panels in the third and fourth columns indicate the lower and upper bounds of an oscillatory domain (Hopf bifurcation points). At the vicinity of the Hopf bifurcation point, **Z** does not converge to a periodic orbit, probably due to numerical error. Therefore, we do not plot *L*_*d*_, –*Z*_*z*_^min^ and –*Z*_*z*_^max^ for such parameter values. We shifted the value of each parameter from the one used in Fig 2 in the main text (S1 Table).
(TIFF)

S5 Fig Dead zone in PRCs of another model for the *Drosophila* circadian clock and dead zone in a repressilator model with the degradation response. (A)-(D) Results for the model proposed by Ueda *et al*. 2001. (A) Time series of cytoplasmic TIM protein TIMc in the absence of light signal for different values of the Michaelis constant for degradation *L*_4_ in Eq. (S7) in S1 Text. (B) Phase shifts Δ*ϕ* as a function of the onset of light signal *t*_*l*_ for different values of *L*_4_. (C), (D) Time series of (top) *TIMc*, (middle) *PER*/*TIM* complex, and (bottom) transcription rate of *Tim*. A light signal is administered at (C) *t*_*l*_ / *T*_*p*_ = 0.2 and (D) *t*_*l*_ / *T*_*p*_ = 0.5 (yellow dotted lines) where *T*_*p*_ is the period of oscillation. (E)-(H) Results for the repressilator model with the degradation response Eq. (S8) in S1 Text. (E) Time series of state variables in the absence of external signal. (F) Phase shift Δ*ϕ* as a function of the onset of external signal *t*_*l*_. Time series of *z* is also plotted (green dotted line) as a reference. (G), (H) Time series of (top) mRNA of X *m*_*x*_, *z* and (bottom) transcription rate of X. An external signal is administered at (G) *t*_*l*_ / *T*_*p*_ = 0.2 and (H) *t*_*l*_ / *T*_*p*_ = 0.7 (yellow dotted lines). *T*_*p*_ = 24. For (F)-(H), *ε*_*l*_ = 0.2 and *T*_*d*_ = 0.5*T*_*p*_/24.
(TIFF)

S6 Fig Phase shift caused by light-induced transcription in the model Eqs (8)–(10). (A) Time series of mRNA *x* and nuclear protein *z* with a light signal (yellow color) in Eqs (8)–(10). (B) Enlargement of the shaded region 4 < *t*/*T*_*p*_ < 5 in (A) to highlight the phase shift. Time series of *x* and *z* with a light signal (perturbed) and those in the absence of a light signal (unperturbed) are shown. Values of parameters are the same as those in Fig 4 in the main text. *T*_*p*_ = 12.67, *ε*_*l*_ = 0.6, *t*_*l*_/*T*_*p*_ = 0.5 and *T*_*d*_ = 0.5*T*_*p*_/24 = 0.3.
(TIFF)

S7 Fig Phase response curves with the saturation of repressor protein transport. (A) Time series of mRNA *x*, cytoplasmic protein *y* and nuclear protein *z* in Eq (8) and (S9) in S1 Text. (B) Phase shift Δ*ϕ* as a function of the onset of light signals *t*_*l*_. Results for different values of *ε*_*l*_ are plotted. *T*_*d*_ = 0.5*T*_*p*_/24 = 0.5. (C) Phase sensitivity *Z*_*x*_. In (B) and (C), the time series of *y* (black broken line) is plotted as a reference. *T*_*p*_ = 24 is the period of oscillation. (D) Phase shift by light-induced transcription. Time series of mRNA *x*, cytoplasmic protein *y* and nuclear protein *z* are shown. For better illustration, two combinations of time series, *x* and *y* in top, *y* and *z* in bottom are presented. The yellow colored regions indicate the light signal at *t*_*l*_/*T*_*p*_ = 0.15. *T*_*p*_ = 24, *ε*_*l*_ = 0.1 and *T*_*d*_ = 0.5*T*_*p*_/24 = 0.5. See S1 Table for the values of reaction parameters.
(TIFF)

S8 Fig Dead zone generated by the saturation of repressor mRNA degradation. (A) Time series of mRNA *x*, cytoplasmic protein *y* and nuclear protein *z* in Eq. (S10) in S1 Text in the absence of light signals. *T*_*p*_ = 24 is the period of oscillation. (B) Phase shift Δ*ϕ* as a function of the onset of light signals *t*_*l*_. Results for different values of *ε*_*l*_ are shown. *T*_*d*_ = 0.5*T*_*p*_/24 = 0.5. (C) Phase sensitivity *Z*_*x*_. In (B) and (C), the time series of *x* (red broken line) is plotted (right y-axis) as a reference. (D)-(G) Time series of mRNA *x* and nuclear protein *z* with the light signal at (D) *t*_*l*_/*T*_*p*_ = 0.1, (E) *t*_*l*_/*T*_*p*_ = 0.47, (F) *t*_*l*_/*T*_*p*_ = 0.64 and (G) *t*_*l*_/*T*_*p*_ = 0.9 (yellow color). *T*_*p*_ = 24, *ε*_*l*_ = 1 and *T*_*d*_ = 0.5*T*_*p*_/24 = 0.5. See S1 Table for the values of reaction parameters.
(TIFF)

S9 Fig Phase shifts by the induction response with the saturation of translation. (A)-(C) Time series of mRNA *x* and nuclear protein *z*. A light signal is administered at (A) *t*/*T*_*p*_ = 0.06, (B) *t*/*T*_*p*_ = 0.4 and (C) *t*/*T*_*p*_ = 0.86 (yellow color) where *T*_*p*_ = 24 is the period of oscillation. Time series of *x* and *z* in the presence of the light signals (perturbed) and those in the absence of a light signal (unperturbed) are shown. Values of parameters in Eqs (8), (10) and (11) are the same as those in Fig 5 in the main text (S1 Table). *ε*_*l*_ = 0.3 and *T*_*d*_ = 0.5.
(TIFF)

S10 Fig Type 0 PRC and entrainment of oscillation to an LD cycle with the induction response. (A) Phase shifts Δ*ϕ* as a function of time *t*_*l*_ at which a light signal is administered. Results for different values of light-induced transcription *ε*_*l*_ in Eq (8) are shown. Time series of mRNA *x* (red broken line) is also plotted (right y-axis) as a reference. (B) Time series of mRNA *x*, nuclear protein *z* and rate of light induced transcription γ_*l*_ under a 12:12 LD cycle. White boxes indicate time windows where light is on and black boxes indicate those where light is off. Values of parameters in Eqs (8), (10) and (11) are the same as those in Fig 5 in the main text (S1 Table). In (A), *T*_*p*_ = 24 and *T*_*d*_ = 2*T*_*p*_/24 = 2. In (B), the free running period is set to 23 with *τ* = 1.08.
(TIFF)

S11 Fig Dependence of the dead zone length on reaction parameters in the model of the induction response Eqs (8), (10) and (11). Dependence on (A) the maximum translation rate γ_1_, (B) protein transport rate γ_2_, (C) the maximum degradation rate of nuclear protein γ_3_, (D) threshold constant *K*_1_ and (E) Michaelis constant for nuclear protein degradation *K*_*m*_. For each parameter, phase sensitivity *Z*_*x*_, time series of mRNA *x*, dead zone length *L*_*d*_, and minimum and maximum values of *Z*_*x*_ (*Z*_*x*_^min^ and *Z*_*x*_^max^, respectively) are shown from left to right. The vertical dotted lines in right two columns indicate the lower and upper bounds of an oscillatory domain (Hopf bifurcation points). At the vicinity of the Hopf bifurcation point, the phase sensitivity **Z** does not converge to a periodic orbit, probably due to numerical error. Therefore, we do not plot *L*_*d*_, *Z*_*x*_^min^ and *Z*_*x*_^max^ for such parameter values. We shifted the value of each parameter from the one used in Fig 5 in the main text (S1 Table).
(TIFF)

S12 Fig Dead zone formation in more complex models with the induction response. (A), (B) Results for the *Per*-*Bmal1*-*Rev-erb* model Eq. (S11) in S1 Text. (A) Time series of *Per*, *Rev-erb* and *Bmal1* mRNAs (top) and proteins (bottom) in the absence of light signals. (B) Phase shift Δ*ϕ* as a function of the onset of the light signal *t*_*l*_ in the *Per*-*Bmal1*-*Rev-erb* model. Time series of *Per* mRNA *x*_*p*_ is also shown as a reference (red dotted line). (C), (D) Results for the repressilator model Eq. (S8a', b', c, d'). (C) Time series of the state variables in the absence of external signals. (D) Phase shift Δ*ϕ* as a function of the onset of the external signal *t*_*l*_. Time series of mRNA for X *m*_*x*_ is shown as a reference (red dotted line). In (B) and (D) *ε*_*l*_ = 0.1 and *T*_*d*_ = 0.5*T*_*p*_/24 = 0.5 with *T*_*p*_ = 24. For the values of other reaction parameters, see the section "Dead zone formation for the induction response in other oscillator models" in S1 Text.
(TIFF)

S13 Fig Reduction of parameter domains for oscillations with stronger translational saturation. (A)-(D) Dependence of the amplitude of mRNA *x* on (A) protein transport rate γ_2_, (B) maximum degradation rate of nuclear protein γ_3_, (C) threshold constant for transcriptional repression *K*_1_ and (D) Michaelis constant for nuclear protein degradation *K*_*m*_ for different values of Michaelis constant for translation *K*_*t*_ in Eqs (8), (10) and (11). Black color indicates that the system converges to a steady state (no oscillation). For better illustration of the effect of translational saturation, the maximum translation rate γ_1_ is scaled as γ_1_ = *cK*_*t*_ where *c* is a constant. We set *c* = 1233.3. With this parametrization, the translation rate for smaller *x* (*x*/*K*_*t*_ ≪ 1) remains constant γ_1_/*K*_*t*_ = *c* for different values of *K*_*t*_. See the section "Parameter domains for oscillation with the saturation of repressor translation" in S1 Text. We shifted the value of each parameter from the one used in Fig 5 in the main text (S1 Table).
(TIFF)

S14 Fig Dead zone generated by the Hill function in translation with a larger threshold constant *K*_*t*_. (A) Phase shift Δ*ϕ* as a function of the onset of light signals *t*_*l*_ with a larger threshold constant *K*_*t*_ in Eq (12) in the main text. The time series of *x* (red broken line) is also plotted as a reference. The horizontal red dotted line plotted against the right y-axis indicates the value of *K*_*t*_. (B), (C) Time series of mRNA *x* and nuclear protein *z* with light signals administered at (B) *t*_*l*_/*T*_*p*_ = 0.42 and (C) *t*_*l*_/*T*_*p*_ = 0.9 (yellow color). (D), (E) Histograms of the dead zone length *L*_*d*_ for (D) *h* = 1 and (E) *h* = 4. Results for 2000 random parameter sets are used. The bin size is 1/24. Parameter values in (A)-(C) are same and listed in S1 Table. *T*_*p*_ = 24, *ε*_*l*_ = 0.1 and *T*_*d*_ = 0.5*T*_*p*_/24 = 0.5 in (A)-(C).
(TIFF)
