# Supplementary material for: A saturated reaction in repressor synthesis creates a daytime dead zone in circadian clocks
Source: PLoS Comput Biol. 2019 Feb 19;15(2):e1006787. doi: 10.1371/journal.pcbi.1006787 (PMC6396941; doi:10.1371/journal.pcbi.1006787)
Supplement: S1 Text — (PDF) [file pcbi.1006787.s001.pdf]

A saturated reaction in repressor synthesis creates a daytime dead zone in circadian clocks

Koichiro Uriu and Hajime Tei

Graduate School of Natural Science and Technology, Kanazawa University, Kakuma-machi, Kanazawa, 920-1192, Japan

S1 Text

Relation between phase shift and phase sensitivity

Here we first derive the equation for phase shift $\Delta\phi$ Eq. (6) in the main text by the phase reduction method following (1). Then, we describe the adjoint method to numerically compute phase sensitivity **Z**.

In general, dynamical system with state variables **x** modeling the circadian clock can be described as

$$\frac{d\mathbf{x}(t)}{dt} = \mathbf{F}(\mathbf{x}(t)), \tag{S1}$$

where **F**(**x**) specifies the time evolution of **x**. In our case, **F**(**x**) describes biochemical reactions in a negative feedback loop in the absence of light signals (i.e. constant dark condition). We assume that the dynamical system Eq. (S1) has a stable limit cycle $\mathbf{x}_{LC}(t)$ in the state space. We then denote dynamics in the presence of perturbation $\mathbf{p}(t, \mathbf{x})$ as

$$\frac{d\mathbf{x}(t)}{dt} = \mathbf{F}(\mathbf{x}(t)) + \mathbf{p}(t, \mathbf{x}(t)). \tag{S2}$$

In the present study, **p** represents the perturbation in biochemical reactions induced by light signals.

We define a phase φ ($0 \le \varphi < 2\pi$) based on the limit cycle of the unperturbed system Eq. (S1) such that φ increases at a constant speed (1):

$$\frac{d\varphi(t)}{dt} = \omega, \tag{S3}$$

where $\omega = 2\pi/T_p$ and T_p is the period of oscillation. We also denote the limit cycle $\mathbf{x}_{LC}(t)$ as a function of φ, $\boldsymbol{\chi}(\varphi) = \mathbf{x}_{LC}(\varphi(t))$. Subsequently, we assign the phase to the state space. If a trajectory $\mathbf{x}(t; \mathbf{x}_0)$ of Eq. (S1) which started at $\mathbf{x}_0$, converges to a point on the limit cycle $\mathbf{x}_{LC}(\varphi_0/\omega + t)$ for $t \to \infty$, we assign the phase φ_0 to $\mathbf{x}_0$. With this definition, we may denote the phase of $\mathbf{x}(t)$ as $\varphi(\mathbf{x}(t))$.

Then, in the presence of weak perturbation ($|\mathbf{p}| << 1$), the time evolution of phase can be described in the first order approximation (1):

$$\frac{d\varphi(t)}{dt} = \omega + \tilde{\mathbf{Z}}(\varphi) \cdot \mathbf{G}(t, \varphi), \tag{S4}$$

where $\tilde{\mathbf{Z}}(\varphi) \equiv \partial\varphi(\chi(\varphi))/\partial\mathbf{x}$ and $\mathbf{G}(t, \varphi) \equiv \mathbf{p}(t, \chi(\varphi))$.

After integrating Eq. (S4), the phase difference $\Delta\phi$ measured in time between perturbed and unperturbed systems with $\gamma_l(t)$ in Eq. (4) in the main text can be approximated as

$$\Delta\phi \approx \frac{\varepsilon T_p}{2\pi}\int_{t_l}^{t_l+T_d} \tilde{\mathbf{Z}}\big(\varphi(t')\big)\cdot\mathbf{G}\big(t',\varphi(t')\big)dt'. \tag{S5}$$

We obtain the phase sensitivity $\mathbf{Z}(t) \equiv (T_p/2\pi)\tilde{\mathbf{Z}}(\varphi(t))$ by numerically solving the following differential equation:

$$\frac{d\mathbf{Z}(t)}{dt} = -L\big(\mathbf{x}_{LC}(t)\big)^{\mathrm{T}}\mathbf{Z}(t), \tag{S6a}$$

with the normalization condition:

$$\mathbf{Z}(t)\cdot\mathbf{F}\big(\mathbf{x}_{LC}(t)\big) = 1, \tag{S6b}$$

where $L(\mathbf{x}_{\mathrm{LC}}(t))$ is the Jacobian around the limit cycle $\mathbf{x}_{\mathrm{LC}}(t)$ of Eq. (S1), $L(\mathbf{x}_{\mathrm{LC}}(t)) = \partial\mathbf{F}/\partial\mathbf{x}|_{\mathbf{x}=\mathbf{x}\mathrm{LC}(t)}$, and T in Eq. (S6a) indicates transpose of a matrix. We used the Euler method to solve Eq. (S6). Note that Eq. (S6) should be solved backward to obtain **Z**, otherwise it diverges because $-L$ has positive eigenvalues for a stable limit cycle.

Dependence of the dead zone length and amplitude of phase sensitivity on reaction parameters in the degradation response

Here, we describe the dependence of the dead zone length L_d and amplitude of phase sensitivity Z_z on each reaction parameter in Eqs. (1-3) for the degradation response (S4 Fig.). In general, the dead zone length L_d depends on a reaction parameter nonmonotonically. There is a peak value of L_d in the oscillatory parameter range between the upper and lower Hopf bifurcation points. The peak of L_d is located near the lower Hopf bifurcation point for the translation rate γ_1 and nuclear transport rate γ_2 (S4A, B Fig.), suggesting that smaller values of these parameters extend the dead zone length. In contrast, for the maximum degradation rate γ_3, the peak of L_d is located near the upper Hopf bifurcation point (S4C Fig.). This result suggests that the faster degradation of nuclear protein favors a longer dead zone. For the threshold for transcriptional repression K_1, L_d peaks near the lower Hopf bifurcation point (S4D Fig.). The condition for the dead

zone $z_{min}/K_1 << 1$ is more likely to be satisfied for a larger value of K_1. However, z_{min} also increases for the larger K_1 (S4D Fig.) and it takes more time for $z(t)$ to return to its original value after light-induced degradation. This slow recovery of $z(t)$ tends to cause prolonged transcription and results in phase delay. Thus, a smaller value of K_1 is more favorable for a longer dead zone.

The amplitude of phase sensitivity $-Z_z$ becomes large near a Hopf bifurcation point (the fourth column of S4 Fig.). At the vicinity of a Hopf bifurcation point, the amplitudes of state variables x, y and z become smaller. It has been reported that the phase of circadian clocks with a smaller amplitude is more sensitive to perturbation, such as light and temperature pulses, than the clocks with a larger amplitude (2, 3). The observation that the magnitude of phase sensitivity is larger near a Hopf bifurcation point is thus consistent with these previous reports.

The time interval where light signals cause phase advances ($-Z_z > 0$) extends for larger values of translation rate γ_1 and/or smaller values of maximum degradation rate of nuclear protein γ_3 (S4A, C Fig.). This is because the decreasing phase of mRNA becomes longer due to higher levels of nuclear protein as γ_1 becomes larger and/or γ_3 becomes smaller. Light-induced degradation of nuclear protein at decreasing phase of mRNA relieves transcriptional repression earlier, resulting in phase advance.

Dead zone formation for the degradation response in other oscillator models

To show the generality of the proposed mechanism for dead zone formation for the degradation response, here we analyze another *Drosophila* circadian clock model and a synthetic oscillator model.

As an alternative model for the *Drosophila* clock, we use the one proposed by Ueda *et al.* 2001 (4). The model includes interlocked feedback loops of PER/TIM and CLOCK/CYCLE (Fig. 2 in (4)). The PER/TIM complex represses their transcription, whereas activates the transcription of *Clock*. The CLOCK/CYCLE complex induces the transcription of *Per* and *Tim*, whereas represses their own transcription. We introduce a light-induced degradation of cytoplasmic TIM protein, Tim_c:

$$\frac{dTim_c}{dt} = S_4 Tim_m - V_1 Per_c Tim_c + V_2 PT_c - \left(D_4 + \gamma_l(t)\right)\frac{Tim_c}{L_4 + Tim_c} - D_0 Tim_c \,, \tag{S7}$$

where Tim_m is the levels of *Tim* mRNA, Per_c is the levels of cytoplasmic PER protein, and PT_c is the levels of the cytoplasmic PER/TIM complex. The first term represents the translation of TIM. The second term represents the PER/TIM complex formation, whereas the third term is for dissociation of the complex. The fourth term represents the nonlinear degradation of TIM protein and the fifth term is its linear degradation. $\gamma_l(t)$ represents the light induced degradation as defined by Eq. (4) in the main text. Dynamics of the other variables are described as in Ueda *et al.* 2001 (4). Also, we use the same parameter values as in Ueda *et al.* 2001.

We confirm that the saturation of TIM degradation lengthens the dead zone by decreasing the values of L_4 in Eq. (S7) (S5A, B Fig.). The minimum value of Tim_c decreases as L_4 becomes smaller (S5A Fig.). To observe how light-induced degradation changes the transcription of *Tim*, we compute the transcription rate determined by PER/TIM, $1/(1+(PT_n/R_2)^r)$ where PT_n is the levels of nuclear PER/TIM, R_2 and r are the threshold and Hill coefficient for repression, respectively, as in Ueda *et al.* 2001. A dead zone is generated when the transcription rate is saturated and a light signal does not further increase it (S5C, D Fig.). Thus, the results derived with the simpler model Eqs. (1-3) in the main text hold for the more complex model of the *Drosophila* clock.

Next, we consider a repressilator model. Although the repressilator was originally proposed as a synthetic oscillator in bacteria (5), the relevance of its regulatory structure to the circadian clock was recently proposed (6). We consider three transcriptional regulators X, Y, and Z. X represses Y, Y represses Z and Z represses X. We denote mRNA levels of X and protein levels of X, Y, and Z as m_x, x, y, and z, respectively. We describe the time evolution of these variables as:

$$\frac{1}{\tau}\frac{dm_x(t)}{dt} = \frac{1}{1+(z(t)/K_1)^n} - m_x(t), \tag{S8a}$$

$$\frac{1}{\tau}\frac{dx(t)}{dt} = \gamma_1 m_x(t) - \gamma_2 x(t), \tag{S8b}$$

$$\frac{1}{\tau}\frac{dy(t)}{dt} = \gamma_3 \frac{1}{1+(x(t)/K_2)^n} - \gamma_4 y(t), \tag{S8c}$$

$$\frac{1}{\tau}\frac{dz(t)}{dt} = \gamma_5 \frac{1}{1+(y(t)/K_3)^n} - \left(\gamma_6 + \gamma_l(t)\right)\frac{z(t)}{K_4 + z(t)}, \tag{S8d}$$

Note that we consider the mRNA of x because we use the same model for the induction response later (see the section "Dead zone formation for the induction response in other oscillator models"). For simplicity, we assume linear degradation of x and y, whereas a saturated degradation of z. γ_l in Eq. (S8d) represents the degradation of z induced by external signals as in the degradation response for the circadian clock. γ_l is defined by Eq. (4) in the main text. We set parameter values as follows: $\gamma_1 = 0.013$, $\gamma_2 = 0.059$, $\gamma_3 = 39.49$, $\gamma_4 = 3.88$, $\gamma_5 = 29.16$, $\gamma_6 = 0.282$, $K_1 = 0.08$, $K_2 = 0.05$, $K_3 = 0.034$, $K_4 = 0.017$, $n = 4$ and $\tau = 0.354$.

The model Eq. (S8) generates a stable limit cycle (S5E Fig.) and a dead zone in a PRC when the levels of z are lower (S5F Fig.). When the dead zone is formed, the transcription rate of x, $1/(1+(z/K_1)^n)$ is saturated (S5G Fig.). In contrast, when a change in the transcription rate occurs, a phase shift is induced (S5H Fig.). Thus, the same conclusion holds for the repressilator model.

Random parameter generation

To check whether a model can create a dead zone in a PRC, we generate parameter sets randomly from uniform distributions and compute phase sensitivity **Z** for limit cycles of those parameter sets. Values of each parameter are chosen randomly from a uniform distribution between 10^{-2} and 10^2. We use a logarithmic scale for choosing values from the uniform distribution. We generate 10^6 parameter sets and examine the linear stability of the steady state. If the Jacobian with a parameter set has complex eigenvalues with a positive real part, we consider that the parameter set can generate a limit cycle. We count the number of such oscillatory parameter sets and compute the fraction over the total 10^6 parameter sets. The fraction should represent how likely a system generates oscillations.

We then randomly select 2000 parameter sets with which the model generates a stable limit cycle. We numerically calculate phase sensitivity $\mathbf{Z}(t)$ of those 2000 limit cycles and check the length of a spanned time window within which $|Z_i(t)| < \theta$ as defined in Eq. (7) in the main text. A phase sensitivity may have several time windows that satisfy this criterion. In such cases, we choose the longest one for statistical analysis shown in S14Fig.

Saturation of transport of repressor protein from the cytoplasm to the nucleus

We examine whether a saturation of repressor protein transport from the cytoplasm to the nucleus can create a dead zone in a PRC of the induction response. We introduce a saturation term of nuclear transport into Eqs. (9) and (10) in the main text as

$$\frac{1}{\tau}\frac{dy(t)}{dt} = \gamma_1 x(t) - \gamma_2 \frac{y(t)}{\kappa_t + y(t)}, \tag{S9a}$$

$$\frac{1}{\tau}\frac{dz(t)}{dt} = \gamma_2 \frac{y(t)}{\kappa_t + y(t)} - \gamma_3 \frac{z(t)}{K_m + z(t)}. \tag{S9b}$$

κ_t much smaller than the peak value of $y(t)$ can saturate the transport in Eq. (S9) (S7A Fig.). The dynamics of mRNA x is described by Eq. (8) in the main text.

The PRC and phase sensitivity of Eqs. (8, S9) shown in S7B, C Fig. do not include an extended dead zone. Figure S7D shows the time series of x, y and z in Eqs. (8, S9) with a light pulse at $t_l/T_p = 0.15$. The increase in mRNA x by the light pulse elevates the levels of cytoplasmic protein y. As expected, the levels of nuclear protein z do not increase immediately after the light pulse due to the saturation of transport. However, the accumulated cytoplasmic protein eventually enters the nucleus, causing a higher peak value of z. The excess amount of nuclear protein thus results in a phase delay.

To further confirm that the saturation of transport cannot create a dead zone, we examine the phase sensitivity of limit cycles of the model Eqs. (8, S9) for randomly generated parameter sets (section "Random parameter generation"). Linear stability analysis around the steady state indicates that 4732 out of 10^6 randomly generated parameter sets (0.47%) can generate limit cycles. This number is ~3-fold lower than that of the non-saturation model Eqs. (8-10) (1.5%) and almost half as that for the translation saturation model Eqs. (8, 10, 11) (0.88%), indicating that saturation of transport reduces parameter domains for oscillations more severely. Moreover, although we numerically compute phase sensitivity Z_x using 2000 parameter sets, their dead zone lengths L_d are less than 1/24. Thus, the saturation of transport is less likely to produce a dead zone. These results also confirm that the saturation of biochemical reactions that is unaccompanied by cancelation of the effect of light signals cannot create a dead zone in the PRC. It is not saturation itself but the unaltered time series of nuclear protein that causes a dead zone.

Dead zone generated by the saturation of repressor mRNA degradation

To test whether the saturation of repressor mRNA degradation can create a dead zone in daytime in the induction response, we consider the following dimensionless equation:

$$\frac{1}{\tau}\frac{dx(t)}{dt} = \frac{\gamma_1}{1+\left(z(t)/K_1\right)^n} + \gamma_l(t) - \gamma_x \frac{x(t)}{K_x + x(t)}, \tag{S10a}$$

$$\frac{1}{\tau}\frac{dy(t)}{dt} = \gamma_1 x(t) - y(t), \tag{S10b}$$

$$\frac{1}{\tau}\frac{dz(t)}{dt} = y(t) - \gamma_2 \frac{z(t)}{K_m + z(t)}, \tag{S10c}$$

where γ_x is the maximum degradation rate and K_x is the Michaelis constant of mRNA degradation. The degradation rate of mRNA can be approximated as ~ γ_x/K_x when x/K_x << 1. Thus, strong saturation (K_x << 1) increases the rate of mRNA degradation when the mRNA abundance is low. Note that because the unit of the degradation rate for the Michaelis-Menten function is different from the one in the liner degradation function, we apply nondimensionalization different from other equations (*e.g.*, Eqs. (8-10) in the main text) to derive Eq. (S10). With the saturation of mRNA degradation, the wave form of mRNA $x(t)$ becomes more pulse-like (S8A Fig.).

In the presence of saturation of mRNA degradation, a dead zone appears when the levels of mRNA x are lower, x/K_x << 1 (S8B, C Fig.). mRNAs induced by a light signal within this time window are quickly degraded due to the sharp increase of the Michaelis-Menten function. Hence, light pulses neither affect the levels of nuclear protein (S8D, G Fig.) nor change the phase of oscillation. In contrast, light signals can cause phase shifts when the levels of mRNA are higher x/K_x >> 1 (S8B, C Fig.). In this regime, the transient increase of mRNA does not affect the speed of degradation due to saturation. A light signal at the increasing phase of mRNA causes phase advance, because it decreases the forthcoming peak of both mRNA and nuclear protein, relieving repression earlier (S8E Fig.). A light signal at the peak or decreasing phases of mRNA results in a phase delay (S8B, C Fig.), as it increases the peak value of nuclear protein, prolonging transcriptional repression (S8F Fig.). In summary, the saturation of repressor mRNA degradation creates a dead zone only when the levels of mRNA are lower.

Dead zone formation for the induction response in other oscillator models

To examine whether saturated translation creates a dead zone in other oscillator models, here we consider an interlocked feedback loop model for the mammalian circadian clock and the repressilator model Eq. (S8).

We first consider the interlocked feedbacks observed in the mammalian circadian clock. The model includes regulations among *Per*, *Bmal1* and *Rev-erb* (7, 8). Free BMAL1 protein induces transcription of *Per* and *Rev-erb* through E-box in their promoter regions. Nuclear PER protein binds to free BMAL1. The BMAL1/PER complex becomes a repressor for *Per* and *Rev-erb* by excluding the binding of free BMAL1 protein to E-box. REV-ERB protein represses the transcription of *Bmal1*. We model the time evolution of mRNA x_i, cytoplasmic protein y_i and nuclear protein z_i ($i \in \{p, b, r\}$). Subscript p indicates that these variables are for *Per*, subscript b for *Bmal1* and r for *Rev-erb*. The time evolution of the mRNA and protein levels is described by the following differential equations:

For *Per*:

$$\frac{1}{\tau}\frac{dx_p(t)}{dt} = \frac{(z_b(t)/K_1)^{n_p}}{1 + (z_b(t)/K_1)^{n_p} + \left(z_b(t)z_p(t)/K_2\right)^{n_p}} + \gamma_l(t) - x_p(t), \tag{S11a}$$

$$\frac{1}{\tau}\frac{dy_p(t)}{dt} = \gamma_1 \frac{x_p(t)}{K_t + x_p(t)} - \gamma_2 y_p(t), \tag{S11b}$$

$$\frac{1}{\tau}\frac{dz_p(t)}{dt} = \gamma_2 y_p(t) - \gamma_3 \frac{z_p(t)}{K_p + z_p(t)}, \tag{S11c}$$

for *Bmal1*:

$$\frac{1}{\tau}\frac{dx_b(t)}{dt} = \frac{1}{1 + (z_r(t)/K_3)^{n_b}} - x_b(t), \tag{S11d}$$

$$\frac{1}{\tau}\frac{dy_b(t)}{dt} = x_b(t) - y_b(t), \tag{S11e}$$

$$\frac{1}{\tau}\frac{dz_b(t)}{dt} = y_b(t) - z_b(t), \tag{S11f}$$

and for *Rev-erb*:

$$\frac{1}{\tau}\frac{dx_r(t)}{dt} = \frac{(z_b(t)/K_4)^{n_r}}{1 + (z_b(t)/K_4)^{n_r} + \left(z_b(t)z_p(t)/K_5\right)^{n_r}} - x_r(t), \tag{S11g}$$

$$\frac{1}{\tau}\frac{dy_r(t)}{dt} = \gamma_4 x_r(t) - \gamma_5 y_r(t), \tag{S11h}$$

$$\frac{1}{\tau}\frac{dz_r(t)}{dt} = \gamma_5 y_r(t) - \gamma_6 \frac{z_r(t)}{K_r + z_r(t)}. \tag{S11i}$$

γ_l in Eq. (S11a) represents the light-induced transcription of *Per*, defined by Eq. (4) in the main text. We introduce the saturation of PER protein translation as in Eq. (S11b). We simplify the dynamics of *Bmal1* by assuming linear translation and degradation of nuclear protein. In addition, we set most parameters in equations for *Bmal1* to unity for simplicity. We use the following parameter values in simulations: γ_1 = 18.5, γ_2 = 0.031, γ_3 = 14.1, γ_4 = 33.0, γ_5 = 2.2, γ_6 = 16.1, K_1 = 0.579, K_2 = 1.83, K_t = 0.015, K_p = 0.025, K_3 = 9.82, K_4 = 0.709, K_5 = 2.0, K_r = 0.782, n_p = 4.0, n_b = 2, n_r = 4 and τ = 1.273.

The model can generate a stable limit cycle (S12A Fig.). The levels of *Per* and *Rev-erb* RNAs peak at similar timing, whereas there is a significant phase difference at protein levels (S12A Fig.). Such phase relations were observed in experiment (9). We confirm that the interlocked feedback model can produce a dead zone when the levels of *Per* mRNA are higher (S12B Fig.).

Next, we consider the repressilator model with the induction response. We modify Eqs. (S8a), (S8b) and (S8d) to include induction by external signals and the saturation of translation as:

$$\frac{1}{\tau}\frac{dm_x(t)}{dt} = \frac{1}{1 + (z(t)/K_1)^n} + \gamma_l(t) - m_x(t), \tag{S8a'}$$

$$\frac{1}{\tau}\frac{dx(t)}{dt} = \gamma_1 \frac{m_x(t)}{K_t + m_x(t)} - \gamma_2 x(t), \tag{S8b'}$$

$$\frac{1}{\tau}\frac{dz(t)}{dt} = \gamma_5 \frac{1}{1 + (y(t)/K_3)^n} - \gamma_6 \frac{z(t)}{K_4 + z(t)}. \tag{S8d'}$$

The equation for protein levels of Y is given by Eq. (S8c). We use the following parameter values in simulations: γ_1 = 0.312, γ_2 = 0.028, γ_3 = 34.48, γ_4 = 2.779, γ_5 = 0.629, γ_6 = 0.035, K_1 = 0.023, K_2 = 5.455, K_3 = 2.121, K_4 = 0.005, K_t = 0.056, n = 4 and τ = 0.92.

The model can generate a stable limit cycle with the above parameter set (S12C Fig.). With the saturation of translation, a dead zone is generated when the levels of m_x are higher (S12D Fig.).

Thus, the saturation of repressor translation can generate a dead zone even in more complex models.

Parameter domains for oscillation with the saturation of repressor translation

Previous studies revealed that nonlinear reaction terms in a negative feedback loop change parameter domains for generating oscillations (10, 11). Saturation of translation tends to make the system less likely to oscillate (10). Indeed, smaller Michaelis constant of translation saturation K_t in Eq. (11) in the main text may lead to dy/d$t \approx \tau(\gamma_1 - \gamma_2 y)$, breaking the feedback loop. To study to what extent saturation of translation in Eq. (11) in the main text reduces the parameter domains for oscillations, we randomly generate 10^6 parameter sets from uniform distributions and examine the fraction of oscillatory parameter sets for both the translation saturation and non-saturation models (section "Random parameter generation"). To determine whether a parameter set can generate a limit cycle, we perform linear stability analysis of Eqs. (8, 10) and (11) in the main text around the steady state. We find that the fraction of oscillatory parameter sets detected in this way is 0.88%. This value is almost half as that for the non-saturation model Eq. (8-10) (1.5%). Thus, the saturation of repressor translation narrows the parameter domains for oscillations.

Then, to clarify how the saturation reduces parameter domains for oscillation, we draw two-dimensional phase diagrams with the amplitude of mRNA x for Eqs. (8, 10) and (11) (S13Fig.). For better comparisons between smaller and larger values of K_t, we fix the ratio $\gamma_1/K_t = c$ in S13 Fig. With this parameterization, translation rate for $x/K_t << 1$ is same among different K_t values, $\sim(\gamma_1/K_t)x = cx$. For γ_2 and K_1, the strong saturation of translation reduces parameter domains for oscillations by both increasing lower bounds and decreasing upper bounds (S13A, C Fig.). In contrast, for γ_3 and K_m, smaller values of K_t mainly decrease the upper bounds of oscillatory domains (S13B, D Fig.), suggesting that slower degradation of nuclear protein and its stronger saturation are required for sustaining oscillation. Given that smaller values of K_m also lengthen the dead zone (S11E

Fig.), the saturation of protein degradation is key to both sustaining oscillation and dead zone formation.

Night-time dead zone generated by the Hill function in translation

We notice that the Hill function in translation Eq. (12) in the main text can create a dead zone when the levels of mRNA x are lower (S14A Fig.). Such dead zone is more likely to be formed when the minimum levels of mRNA x is much lower than the threshold K_t in the Hill function of Eq. (12). A Hill function realizes a translational switch with a threshold K_t. If the levels of mRNA x are lower than K_t and light induction is weak, x does not surpass K_t and translation does not occur. Hence, when the levels of mRNA are low, the Hill function can cancel the influence of the mRNA induction by light signals (S14B, C Fig.). This result further indicates that the cancelation of the influence of mRNA induction by light signals generates a dead zone in the PRC.

Subsequently, we study the dead zone formation with randomly generated parameter sets. For this, we fix the Hill coefficient h in Eq. (12) as $h = 4$. As before, we generate 10^6 parameter sets randomly from uniform distributions and check the linear stability of the steady state (section "Random parameter generation"). This analysis finds that 36174 out of 10^6 (~3.6%) parameter sets are oscillatory. In contrast, the fraction of oscillatory parameter sets for the linear translation model Eq. (8-10) in the main text is ~1.5%. Thus, the Hill function in translation allows the system to be more oscillatory than the linear translation process Eq. (9). Furthermore, the dead zone length L_d distributes more broadly for $h = 4$ than $h = 1$ (S14D, E Fig.). Out of 2000 parameter sets used for numerical computation of the phase sensitivity Z_x, 17.7% (354/2000) create dead zones of which length L_d is longer than 1/24. 30 parameter sets out of 354 generate dead zones when the levels of mRNA are higher than K_t as shown in Fig. 7A. Remaining 324 form dead zones when the levels of mRNA are lower than K_t as shown in S14A Fig. Our analysis on the Hill function indicates that the type of nonlinear functions in translation can determine at which phase of oscillation a dead zone is generated.

SUPPLEMENTARY REFERENCES

1. Kuramoto Y (1984) *Chemical Oscillations, Waves, and Turbulence* (Springer-Verlag, Berlin, Heidelberg).

2. Abraham U, *et al.* (2010) Coupling governs entrainment range of circadian clocks. *Mol Syst Biol* 6:438.
3. Vitaterna MH, *et al.* (2006) The mouse Clock mutation reduces circadian pacemaker amplitude and enhances efficacy of resetting stimuli and phase-response curve amplitude. *Proc Natl Acad Sci U S A* 103(24):9327-9332.
4. Ueda HR, Hagiwara M, & Kitano H (2001) Robust oscillations within the interlocked feedback model of Drosophila circadian rhythm. *J Theor Biol* 210(4):401-406.
5. Elowitz MB & Leibler S (2000) A synthetic oscillatory network of transcriptional regulators. *Nature* 403(6767):335-338.
6. Pett JP, Korencic A, Wesener F, Kramer A, & Herzel H (2016) Feedback Loops of the Mammalian Circadian Clock Constitute Repressilator. *PLoS Comput Biol* 12(12):e1005266.
7. Takahashi JS (2017) Transcriptional architecture of the mammalian circadian clock. *Nature reviews. Genetics* 18(3):164-179.
8. Preitner N, *et al.* (2002) The orphan nuclear receptor REV-ERBalpha controls circadian transcription within the positive limb of the mammalian circadian oscillator. *Cell* 110(2):251-260.
9. Narumi R, *et al.* (2016) Mass spectrometry-based absolute quantification reveals rhythmic variation of mouse circadian clock proteins. *Proc Natl Acad Sci U S A* 113(24):E3461-3467.
10. Kurosawa G & Iwasa Y (2002) Saturation of enzyme kinetics in circadian clock models. *J Biol Rhythms* 17(6):568-577.
11. Kurosawa G, Mochizuki A, & Iwasa Y (2002) Comparative study of circadian clock models, in search of processes promoting oscillation. *J Theor Biol* 216(2):193-208.
