# Supplementary material for: A saturated reaction in repressor synthesis creates a daytime dead zone in circadian clocks
Source: PLoS Comput Biol. 2019 Feb 19;15(2):e1006787. doi: 10.1371/journal.pcbi.1006787 (PMC6396941; doi:10.1371/journal.pcbi.1006787)
Supplement: S1 Table — (PDF) [file pcbi.1006787.s002.pdf]

S1 Table. Values of reaction parameters in the models

Eqs. (1-3)

	γ_1	γ_2	γ_3	K_1	K_m	n	τ
Fig. 2	0.75	0.37	0.6	0.094	0.001	4	0.2853
Fig. 3	0.75	0.37	0.6	0.094	0.0005<br>0.0025<br>0.005	4	0.2982<br>0.2606<br>0.2374

Eqs. (8-10) and Eqs. (8, 10, 11)

	γ_1	γ_2	γ_3	K_1	K_t	K_m	n	τ
Fig. 4	18.5	0.031	14.1	1.83	-	0.025	4	1.034
Fig. 5	18.5	0.031	14.1	1.83	0.015	0.025	4	1.034
Fig. 6	18.5	0.031	14.1	1.83	0.01<br>0.06<br>0.1	0.025	4	1.089<br>0.8588<br>0.7979

Eqs. (8, 10, 12)

	γ_1	γ_2	γ_3	K_1	K_t	K_m	n	h	τ
Fig. 7	18.5	0.031	14.1	1.83	0.015	0.053	4	1<br>2<br>4	0.912<br>0.9619<br>0.9872
Fig. S14	0.66	0.156	0.062	0.114	0.22	0.0645	4	4	0.7675

Eqs. (8, S9a,b)

	γ_1	γ_2	γ_3	K_1	κ_t	K_m	n	τ
Fig. S7	77.5	6.56	5.19	0.114	0.07	0.023	4	0.0943

Eq. (S10)

	γ_1	γ_x	γ_2	K_1	K_x	K_m	n	τ
Fig. S8	6.55	4.4	1.04	0.837	0.035	0.061	4	0.1729
