# Supplementary figures and images for: A saturated reaction in repressor synthesis creates a daytime dead zone in circadian clocks
Source: PLoS Comput Biol. 2019 Feb 19;15(2):e1006787. doi: 10.1371/journal.pcbi.1006787 (PMC6396941; doi:10.1371/journal.pcbi.1006787)

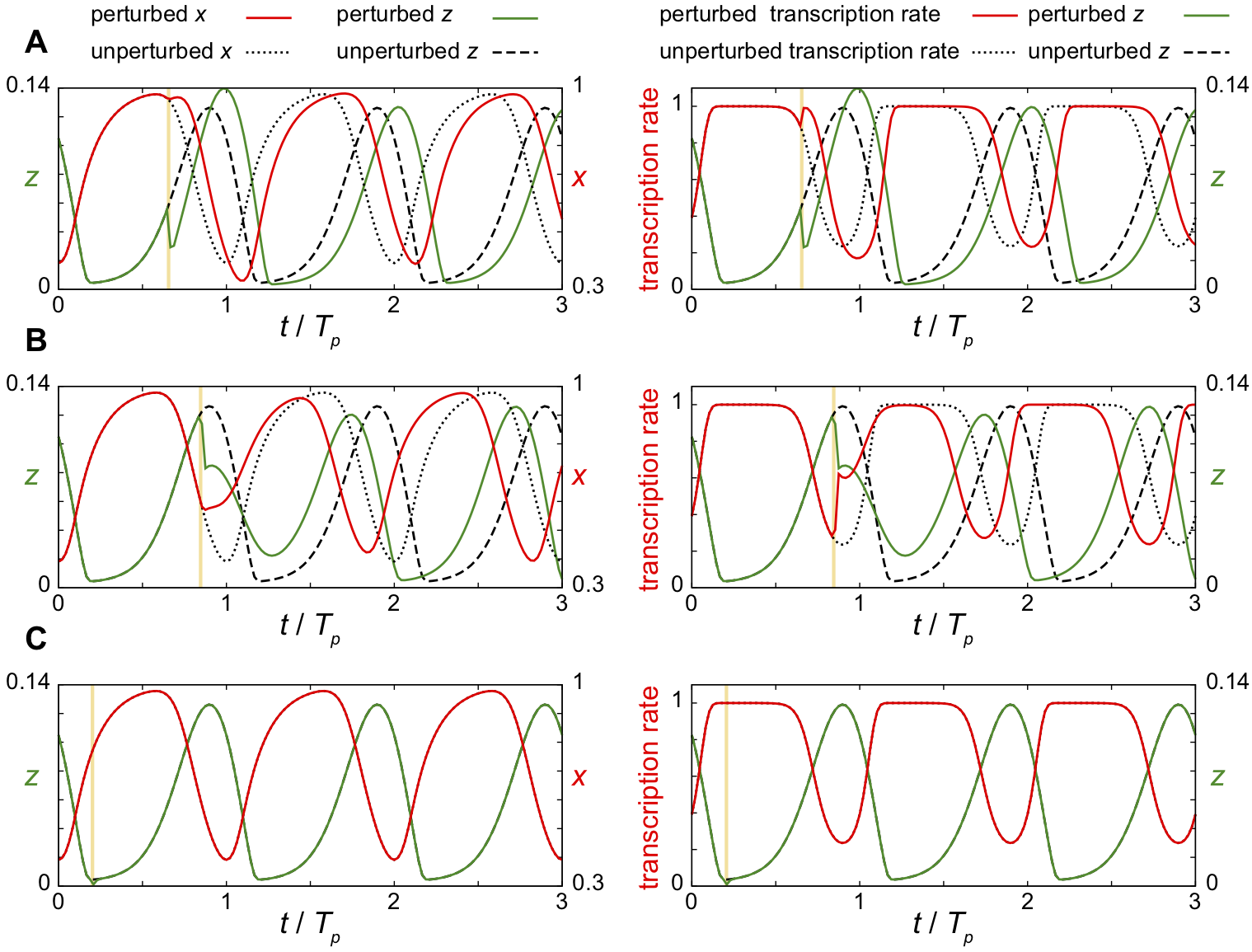

Supplement: S1 Fig — (A)-(C) Time series of mRNA x and nuclear protein z (left) and that of transcription rate 1/(1+(z/K1)n) in Eq (1) in the main text (right) for the degradation response. Light is administered at (A) t/Tp = 0.65, (B) t/Tp = 0.85 and (C) t/Tp = 0.2 where Tp = 24 is the period of oscillation. Time series of x, z and transcription rate with a light signal (perturbed) and those in the absence of a light signal (unperturbed) are shown. Values of reaction parameters in Eqs (1)–(3) are the same as those in Fig 2 in the main text (S1 Table). εl = 0.3 and Td = 0.5Tp/24 = 0.5. (TIFF) [file pcbi.1006787.s003.tiff]

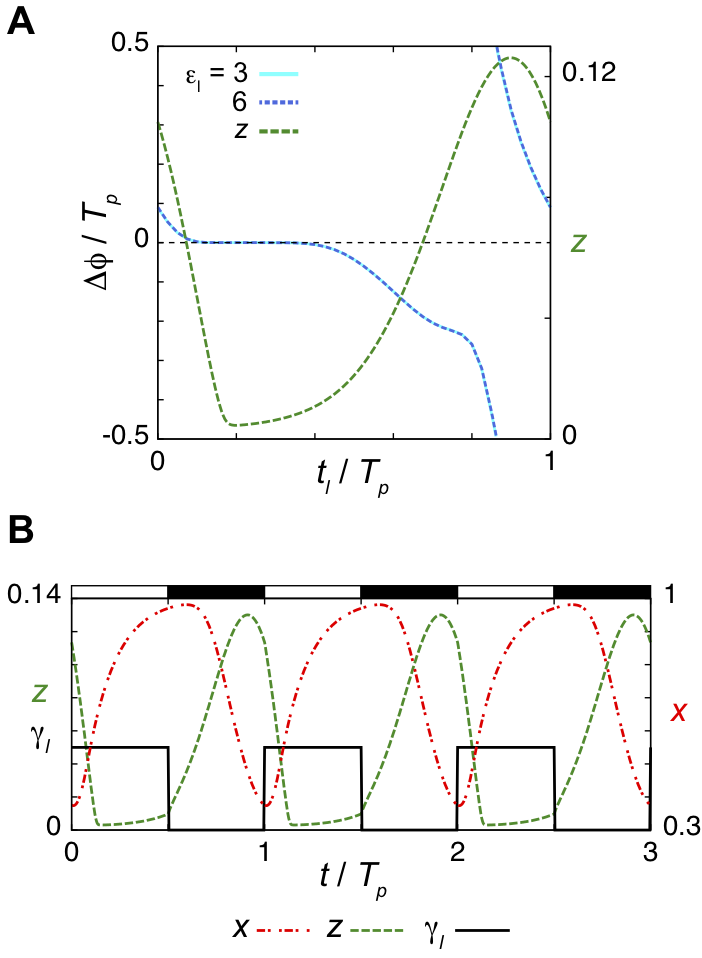

Supplement: S2 Fig — (A) Phase shifts Δϕ as a function of time tl at which a light signal is administered. Results for different values of rate of light-induced degradation εl are plotted. Note that the results for εl = 3 and 6 are overlapped. Time series of nuclear protein z (green broken line) is also shown as a reference. Td = 0.5Tp/24 = 0.5. (B) Time series of mRNA x, nuclear protein z and light-induced degradation γl under a 12:12 LD cycle. White boxes indicate time windows where light is on and black boxes indicate those where light is off. The free running period is set to 24.3 with τ = 0.2818 in Eqs (1)–(3). Values of parameters in Eqs (1)–(3) are the same as those in Fig 2 (S1 Table). (TIFF) [file pcbi.1006787.s004.tiff]

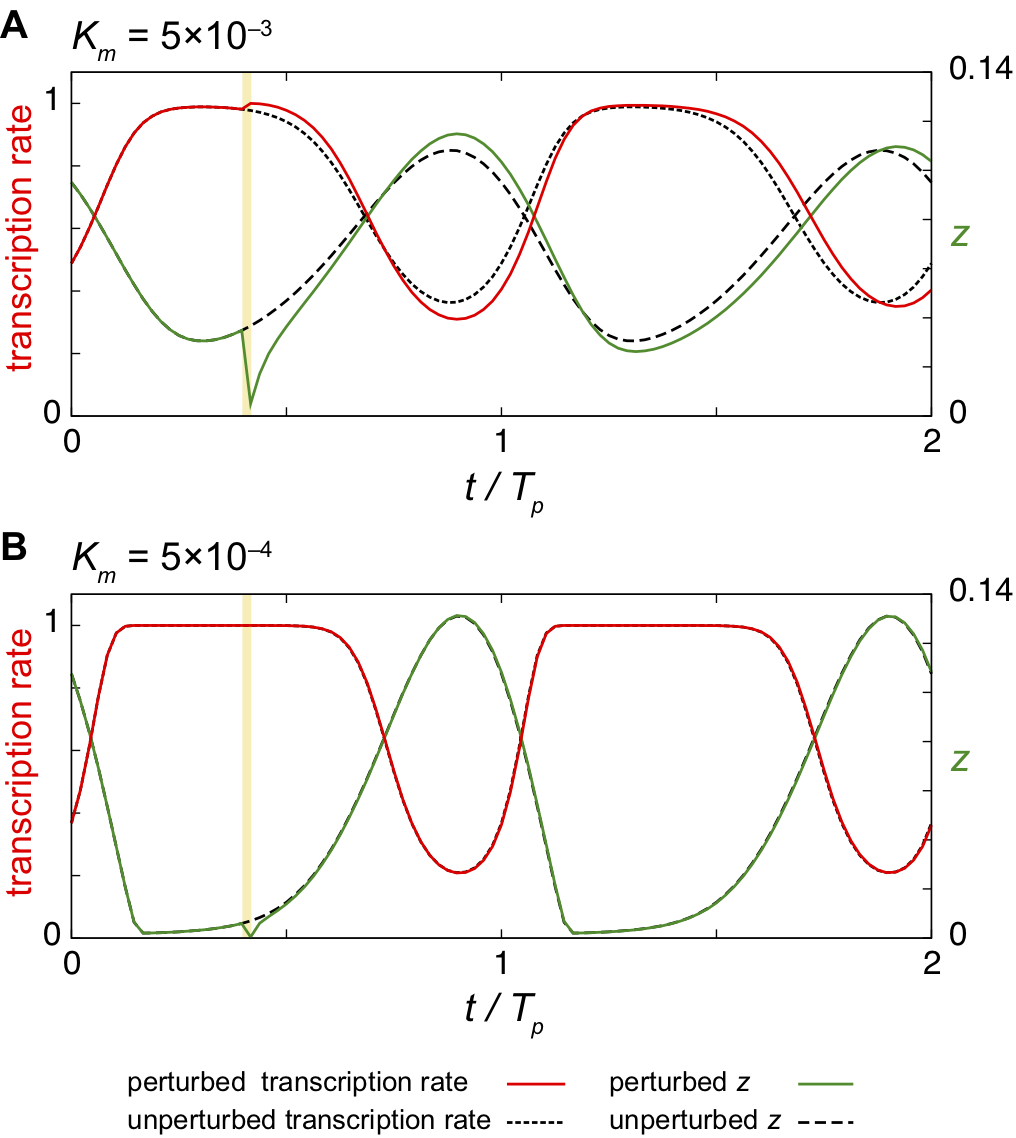

Supplement: S3 Fig — (A), (B) Time series of nuclear protein z and that of transcription rate 1/(1+(z/K1)n) in Eq (1) in the main text for the different values of Michaelis constant Km for nuclear protein degradation. (A) Km = 5×10^−3^ and (B) Km = 5×10^−4^. Light is administered at t/Tp = 0.4 where Tp = 24 is the period of oscillation. Time series of z and transcription rate with a light signal (perturbed) and those in the absence of a light signal (unperturbed) are shown. Values of the other reaction parameters in Eqs (1)–(3) are the same as those in Fig 3 (S1 Table). εl = 0.6 and Td = 0.5Tp/24 = 0.5. (TIFF) [file pcbi.1006787.s005.tiff]

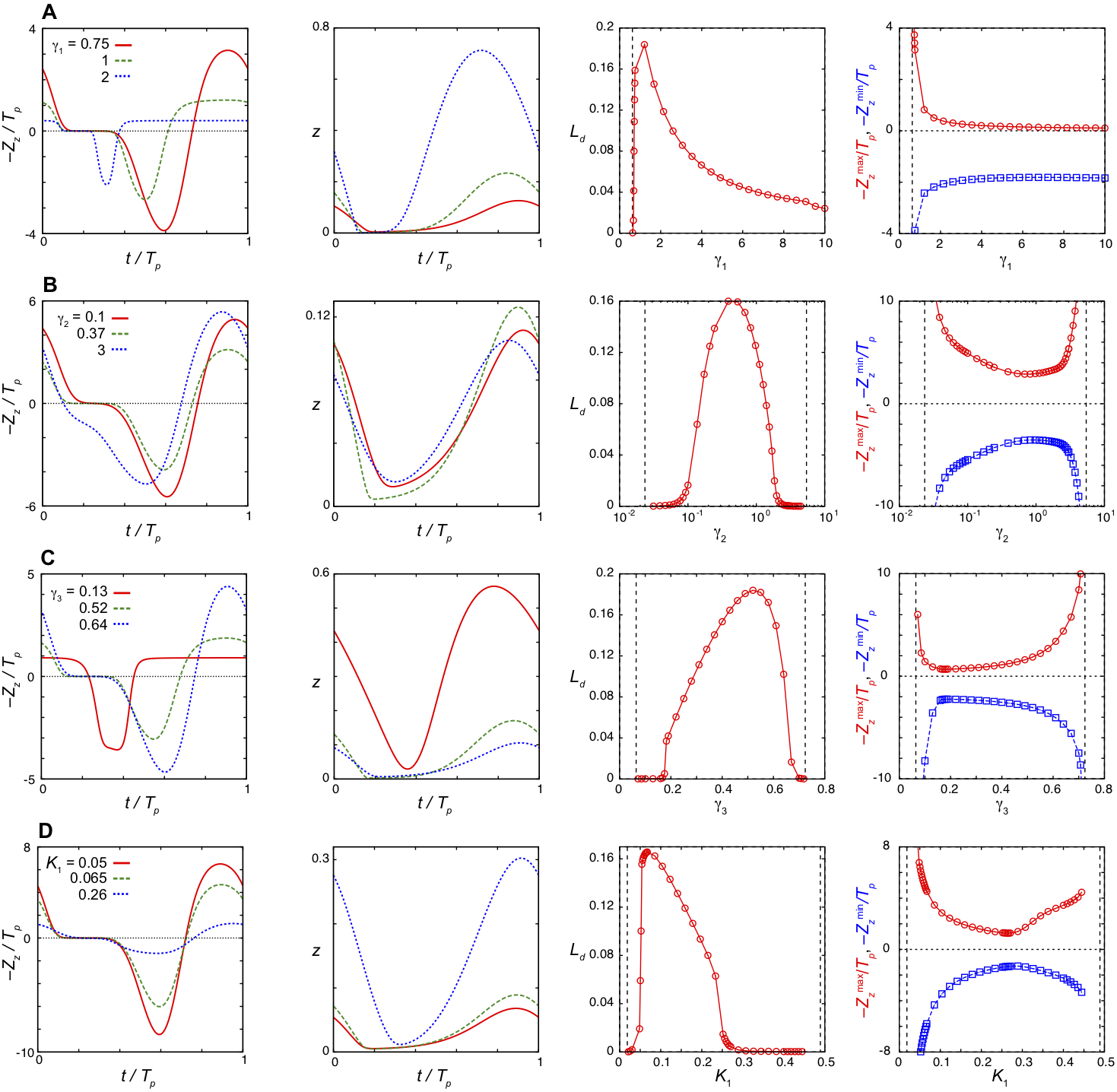

Supplement: S4 Fig — Dependence on (A) translation rate γ1, (B) protein transport rate γ2, (C) degradation rate of nuclear protein γ3 and (D) threshold constant for transcriptional repression K1. The first column shows the phase sensitivity –Zz for different values of the focal parameter. The second column shows the time series of nuclear protein z. Line types correspond to those in the first column. The third column shows the dead zone length Ld as a function of the focal parameter. The fourth column shows minimum and maximum values of the phase sensitivity –Zzmin and –Zzmax, respectively. The vertical dotted lines in panels in the third and fourth columns indicate the lower and upper bounds of an oscillatory domain (Hopf bifurcation points). At the vicinity of the Hopf bifurcation point, Z does not converge to a periodic orbit, probably due to numerical error. Therefore, we do not plot Ld, –Zzmin and –Zzmax for such parameter values. We shifted the value of each parameter from the one used in Fig 2 in the main text (S1 Table). (TIFF) [file pcbi.1006787.s006.tiff]

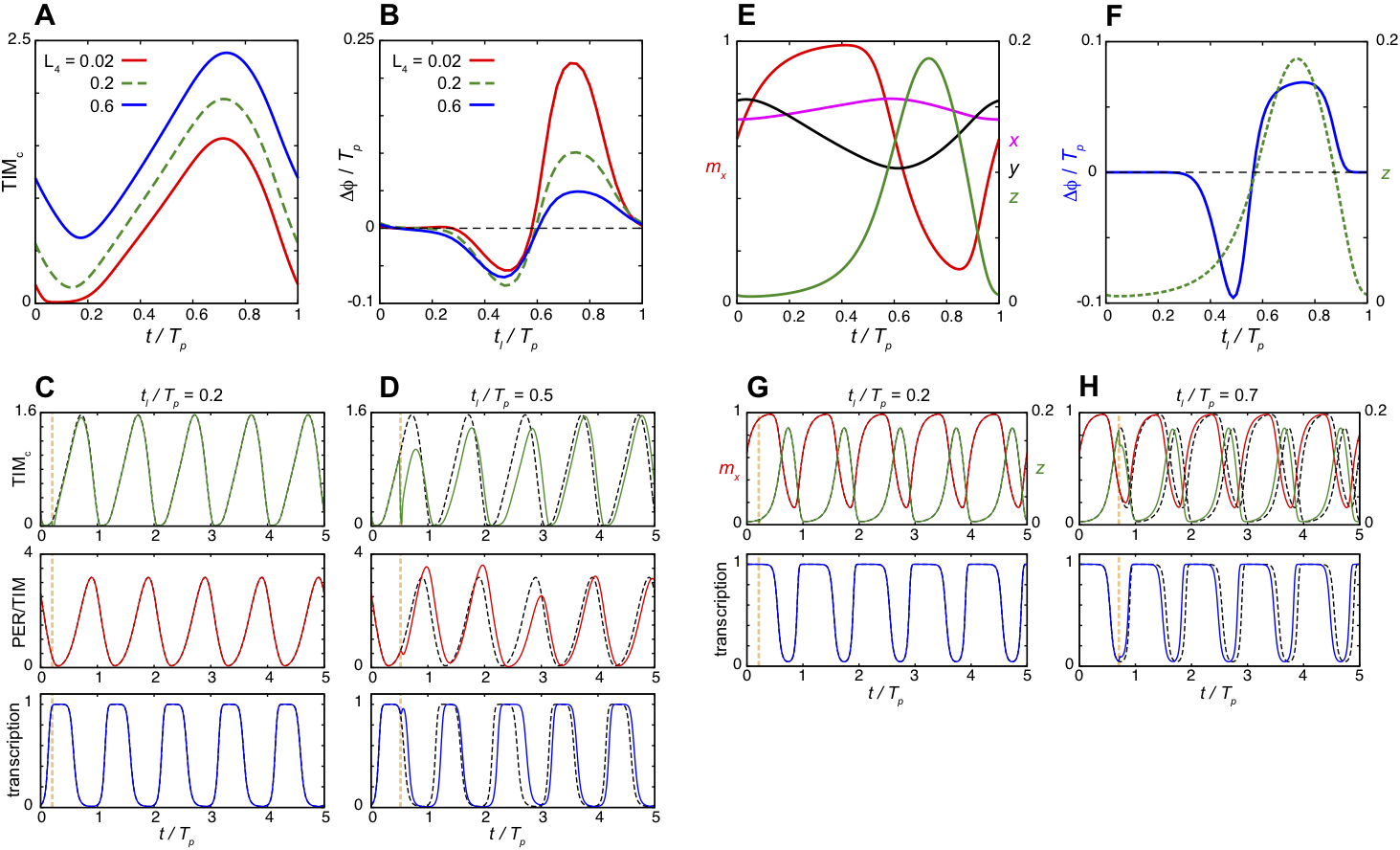

Supplement: S5 Fig — (A)-(D) Results for the model proposed by Ueda et al. 2001. (A) Time series of cytoplasmic TIM protein TIMc in the absence of light signal for different values of the Michaelis constant for degradation L4 in Eq. (S7) in S1 Text. (B) Phase shifts Δϕ as a function of the onset of light signal tl for different values of L4. (C), (D) Time series of (top) TIMc, (middle) PER/TIM complex, and (bottom) transcription rate of Tim. A light signal is administered at (C) tl / Tp = 0.2 and (D) tl / Tp = 0.5 (yellow dotted lines) where Tp is the period of oscillation. (E)-(H) Results for the repressilator model with the degradation response Eq. (S8) in S1 Text. (E) Time series of state variables in the absence of external signal. (F) Phase shift Δϕ as a function of the onset of external signal tl. Time series of z is also plotted (green dotted line) as a reference. (G), (H) Time series of (top) mRNA of X mx, z and (bottom) transcription rate of X. An external signal is administered at (G) tl / Tp = 0.2 and (H) tl / Tp = 0.7 (yellow dotted lines). Tp = 24. For (F)-(H), εl = 0.2 and Td = 0.5Tp/24. (TIFF) [file pcbi.1006787.s007.tiff]

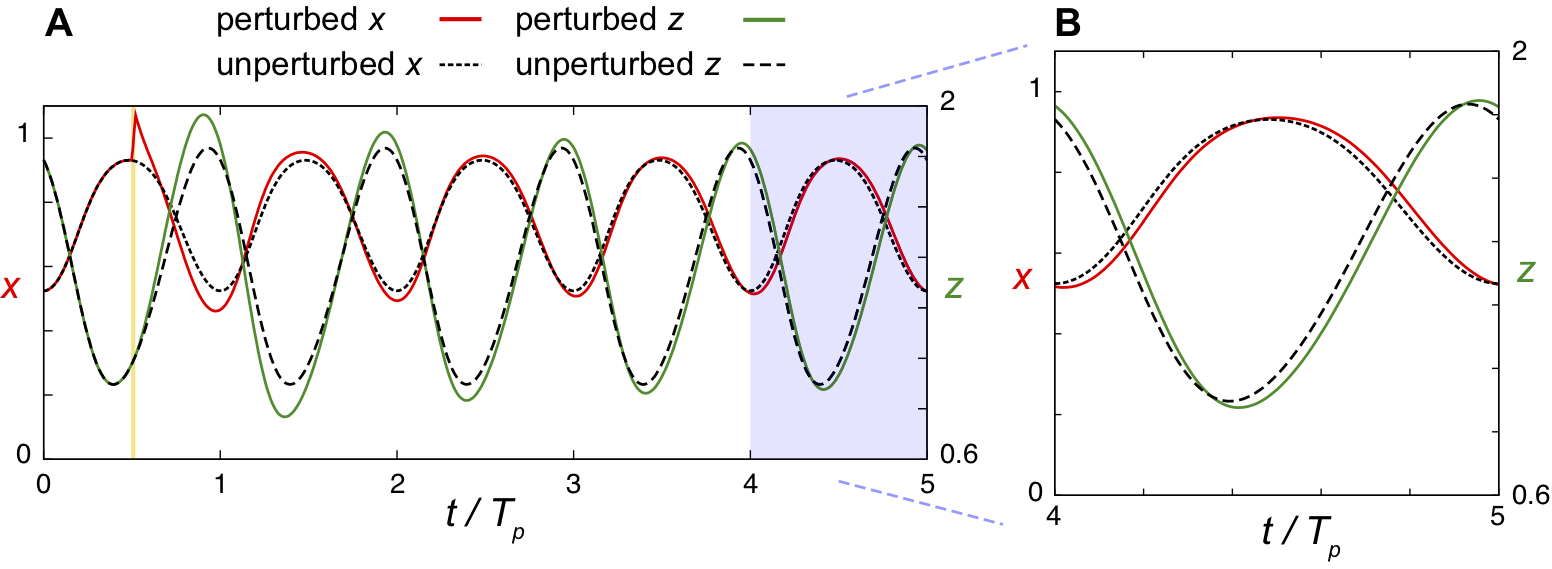

Supplement: S6 Fig — (A) Time series of mRNA x and nuclear protein z with a light signal (yellow color) in Eqs (8)–(10). (B) Enlargement of the shaded region 4 < t/Tp < 5 in (A) to highlight the phase shift. Time series of x and z with a light signal (perturbed) and those in the absence of a light signal (unperturbed) are shown. Values of parameters are the same as those in Fig 4 in the main text. Tp = 12.67, εl = 0.6, tl/Tp = 0.5 and Td = 0.5Tp/24 = 0.3. (TIFF) [file pcbi.1006787.s008.tiff]

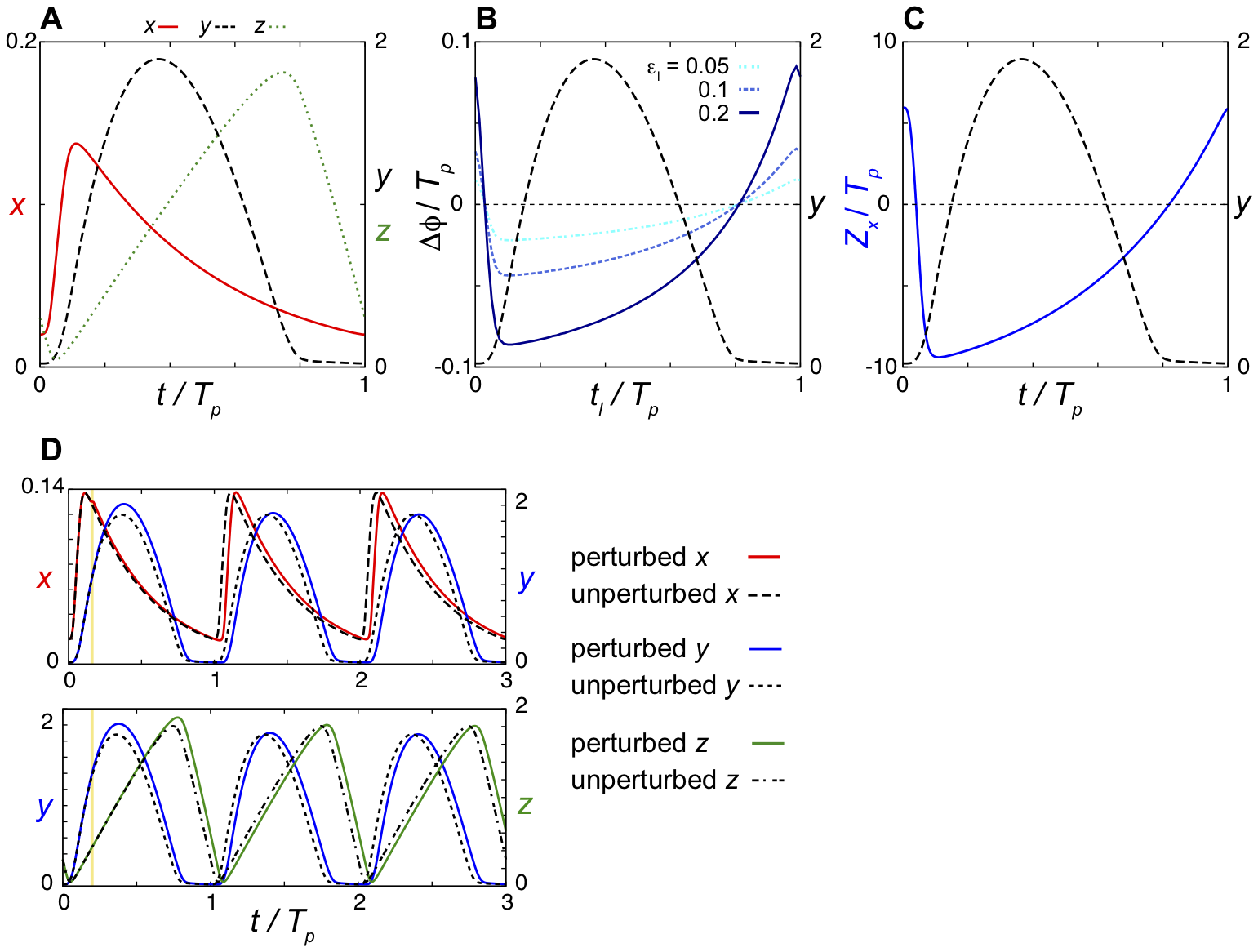

Supplement: S7 Fig — (A) Time series of mRNA x, cytoplasmic protein y and nuclear protein z in Eq (8) and (S9) in S1 Text. (B) Phase shift Δϕ as a function of the onset of light signals tl. Results for different values of εl are plotted. Td = 0.5Tp/24 = 0.5. (C) Phase sensitivity Zx. In (B) and (C), the time series of y (black broken line) is plotted as a reference. Tp = 24 is the period of oscillation. (D) Phase shift by light-induced transcription. Time series of mRNA x, cytoplasmic protein y and nuclear protein z are shown. For better illustration, two combinations of time series, x and y in top, y and z in bottom are presented. The yellow colored regions indicate the light signal at tl/Tp = 0.15. Tp = 24, εl = 0.1 and Td = 0.5Tp/24 = 0.5. See S1 Table for the values of reaction parameters. (TIFF) [file pcbi.1006787.s009.tiff]

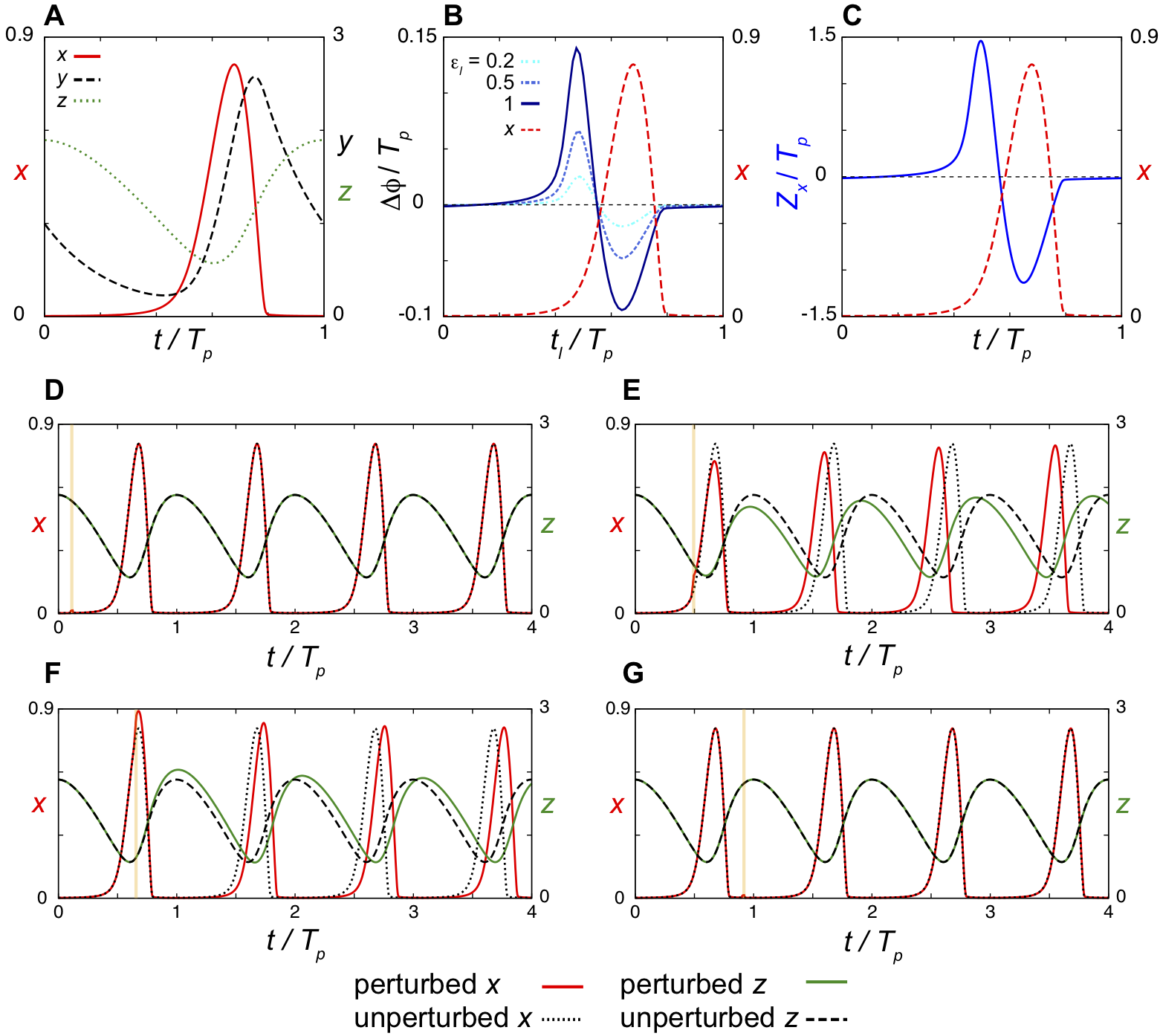

Supplement: S8 Fig — (A) Time series of mRNA x, cytoplasmic protein y and nuclear protein z in Eq. (S10) in S1 Text in the absence of light signals. Tp = 24 is the period of oscillation. (B) Phase shift Δϕ as a function of the onset of light signals tl. Results for different values of εl are shown. Td = 0.5Tp/24 = 0.5. (C) Phase sensitivity Zx. In (B) and (C), the time series of x (red broken line) is plotted (right y-axis) as a reference. (D)-(G) Time series of mRNA x and nuclear protein z with the light signal at (D) tl/Tp = 0.1, (E) tl/Tp = 0.47, (F) tl/Tp = 0.64 and (G) tl/Tp = 0.9 (yellow color). Tp = 24, εl = 1 and Td = 0.5Tp/24 = 0.5. See S1 Table for the values of reaction parameters. (TIFF) [file pcbi.1006787.s010.tiff]

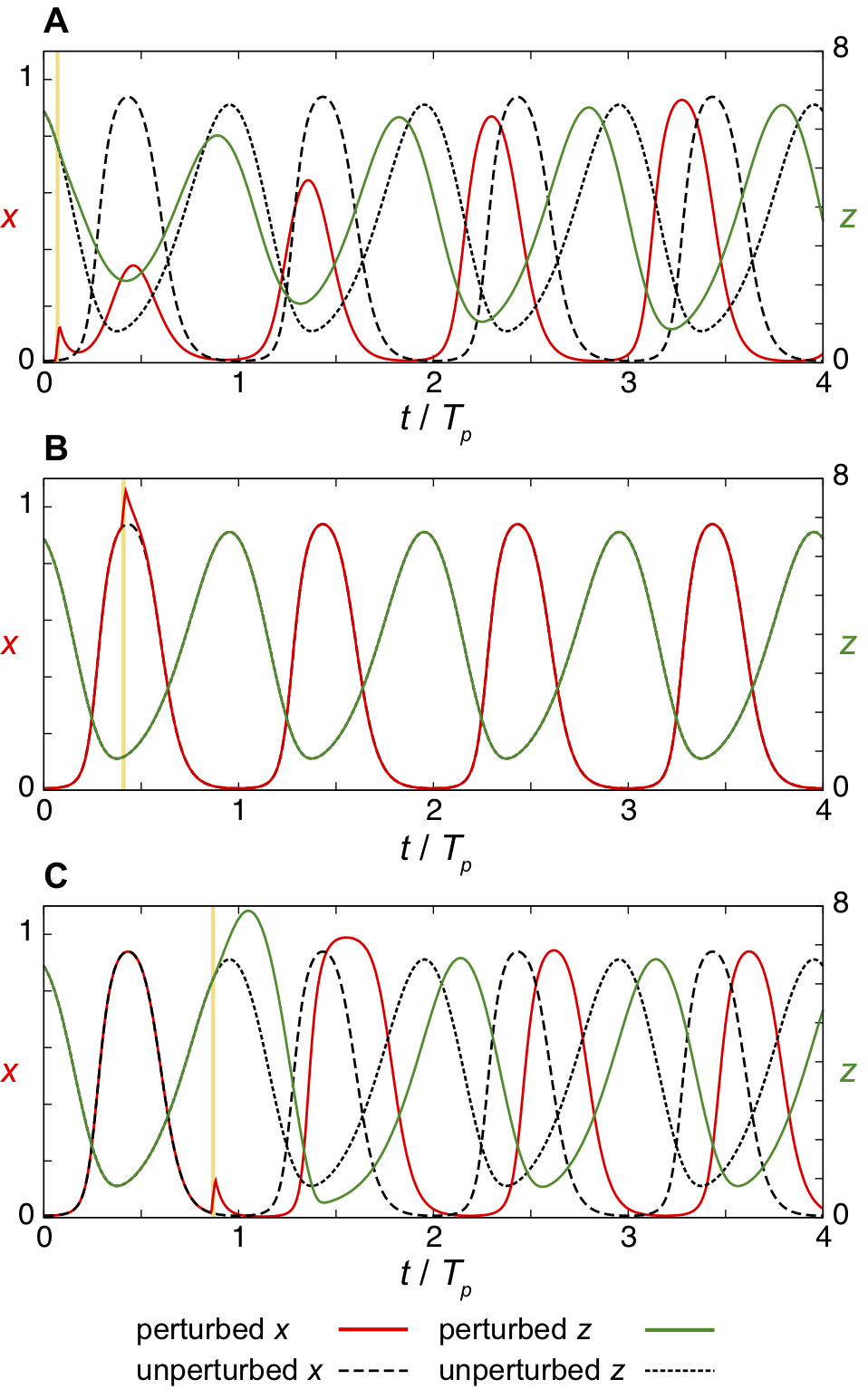

Supplement: S9 Fig — (A)-(C) Time series of mRNA x and nuclear protein z. A light signal is administered at (A) t/Tp = 0.06, (B) t/Tp = 0.4 and (C) t/Tp = 0.86 (yellow color) where Tp = 24 is the period of oscillation. Time series of x and z in the presence of the light signals (perturbed) and those in the absence of a light signal (unperturbed) are shown. Values of parameters in Eqs (8), (10) and (11) are the same as those in Fig 5 in the main text (S1 Table). εl = 0.3 and Td = 0.5. (TIFF) [file pcbi.1006787.s011.tiff]

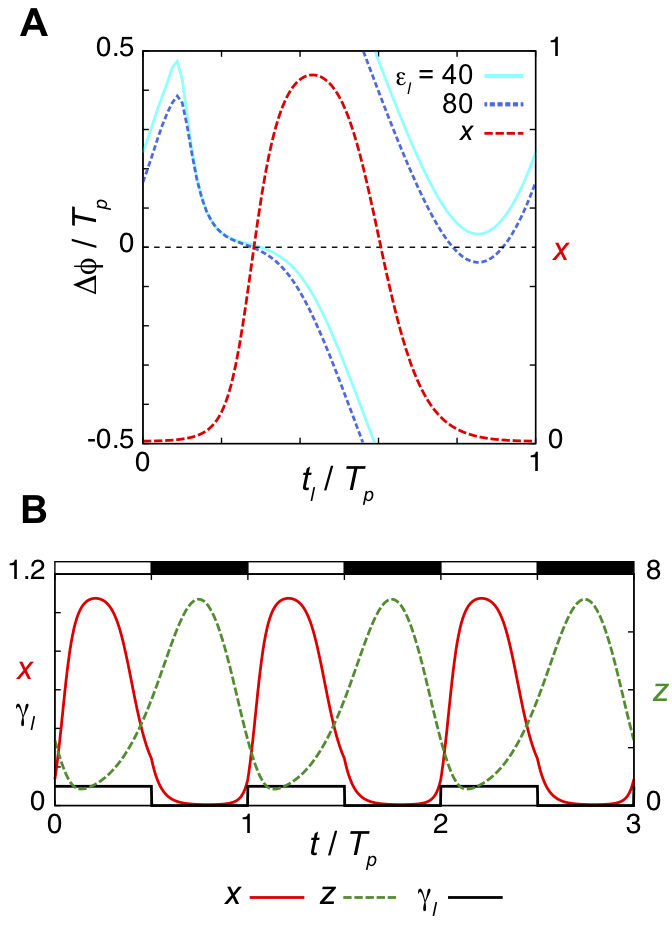

Supplement: S10 Fig — (A) Phase shifts Δϕ as a function of time tl at which a light signal is administered. Results for different values of light-induced transcription εl in Eq (8) are shown. Time series of mRNA x (red broken line) is also plotted (right y-axis) as a reference. (B) Time series of mRNA x, nuclear protein z and rate of light induced transcription γl under a 12:12 LD cycle. White boxes indicate time windows where light is on and black boxes indicate those where light is off. Values of parameters in Eqs (8), (10) and (11) are the same as those in Fig 5 in the main text (S1 Table). In (A), Tp = 24 and Td = 2Tp/24 = 2. In (B), the free running period is set to 23 with τ = 1.08. (TIFF) [file pcbi.1006787.s012.tiff]

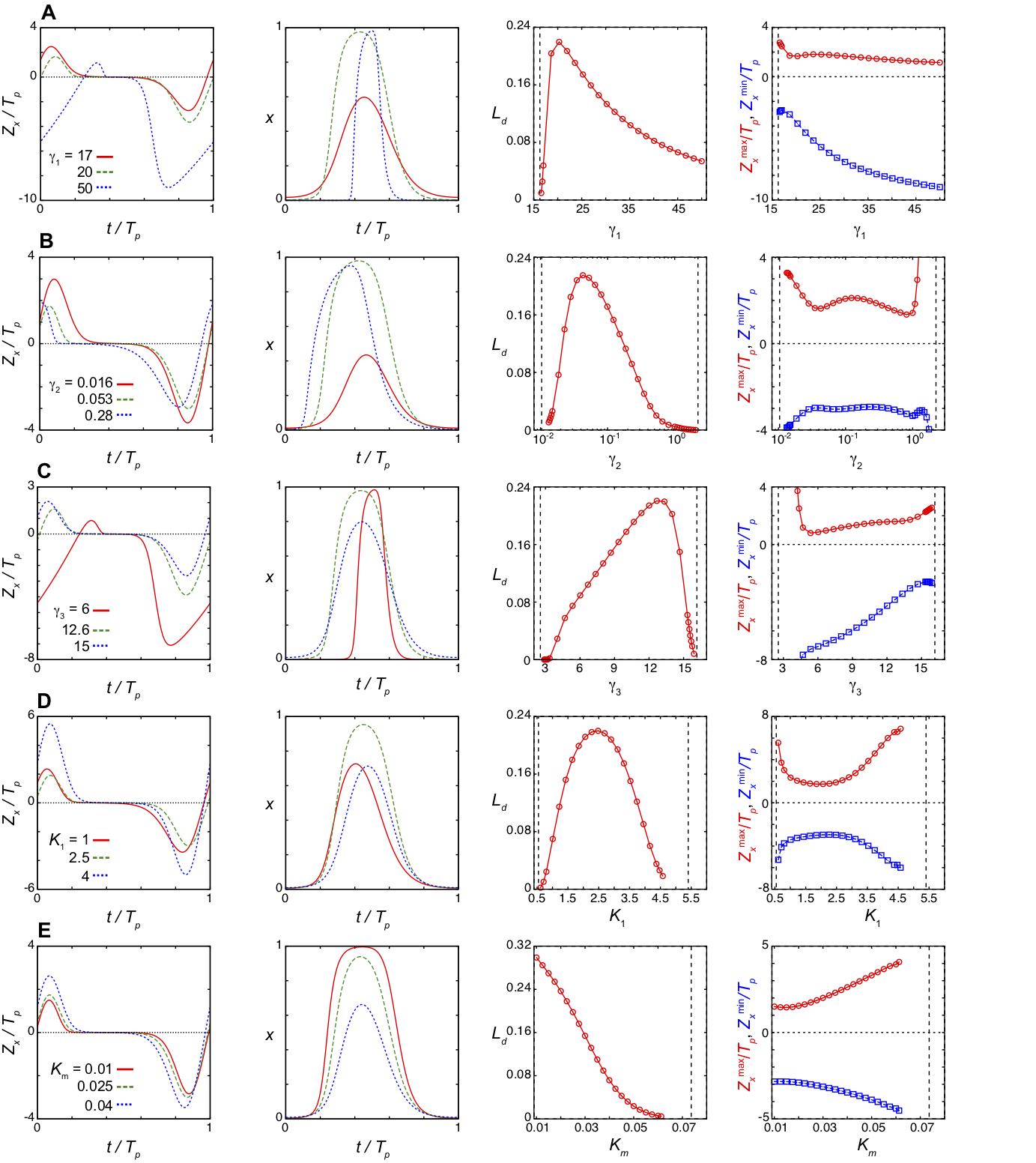

Supplement: S11 Fig — Dependence on (A) the maximum translation rate γ1, (B) protein transport rate γ2, (C) the maximum degradation rate of nuclear protein γ3, (D) threshold constant K1 and (E) Michaelis constant for nuclear protein degradation Km. For each parameter, phase sensitivity Zx, time series of mRNA x, dead zone length Ld, and minimum and maximum values of Zx (Zxmin and Zxmax, respectively) are shown from left to right. The vertical dotted lines in right two columns indicate the lower and upper bounds of an oscillatory domain (Hopf bifurcation points). At the vicinity of the Hopf bifurcation point, the phase sensitivity Z does not converge to a periodic orbit, probably due to numerical error. Therefore, we do not plot Ld, Zxmin and Zxmax for such parameter values. We shifted the value of each parameter from the one used in Fig 5 in the main text (S1 Table). (TIFF) [file pcbi.1006787.s013.tiff]

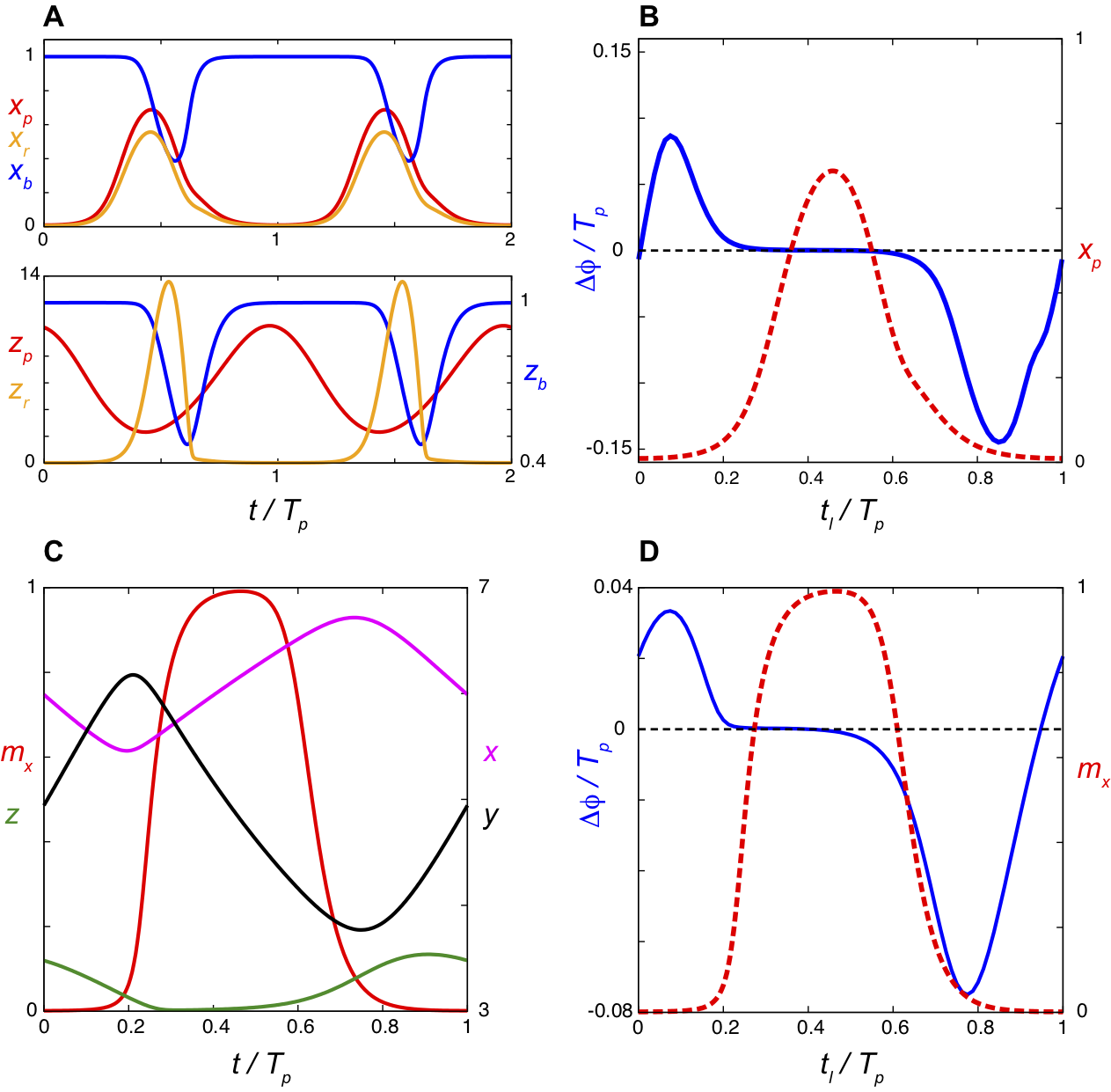

Supplement: S12 Fig — (A), (B) Results for the Per-Bmal1-Rev-erb model Eq. (S11) in S1 Text. (A) Time series of Per, Rev-erb and Bmal1 mRNAs (top) and proteins (bottom) in the absence of light signals. (B) Phase shift Δϕ as a function of the onset of the light signal tl in the Per-Bmal1-Rev-erb model. Time series of Per mRNA xp is also shown as a reference (red dotted line). (C), (D) Results for the repressilator model Eq. (S8a', b', c, d'). (C) Time series of the state variables in the absence of external signals. (D) Phase shift Δϕ as a function of the onset of the external signal tl. Time series of mRNA for X mx is shown as a reference (red dotted line). In (B) and (D) εl = 0.1 and Td = 0.5Tp/24 = 0.5 with Tp = 24. For the values of other reaction parameters, see the section "Dead zone formation for the induction response in other oscillator models" in S1 Text. (TIFF) [file pcbi.1006787.s014.tiff]

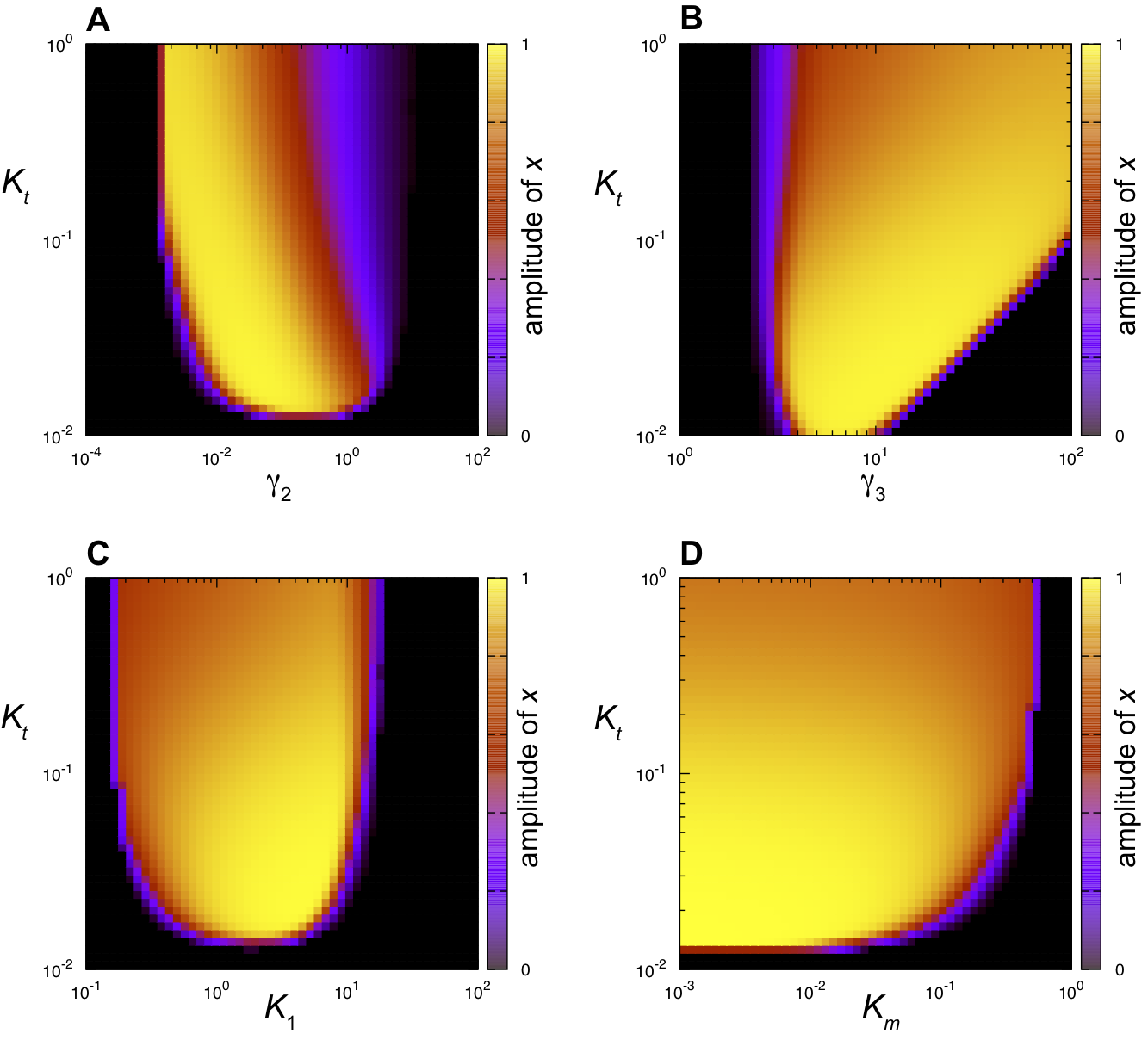

Supplement: S13 Fig — (A)-(D) Dependence of the amplitude of mRNA x on (A) protein transport rate γ2, (B) maximum degradation rate of nuclear protein γ3, (C) threshold constant for transcriptional repression K1 and (D) Michaelis constant for nuclear protein degradation Km for different values of Michaelis constant for translation Kt in Eqs (8), (10) and (11). Black color indicates that the system converges to a steady state (no oscillation). For better illustration of the effect of translational saturation, the maximum translation rate γ1 is scaled as γ1 = cKt where c is a constant. We set c = 1233.3. With this parametrization, the translation rate for smaller x (x/Kt ≪ 1) remains constant γ1/Kt = c for different values of Kt. See the section "Parameter domains for oscillation with the saturation of repressor translation" in S1 Text. We shifted the value of each parameter from the one used in Fig 5 in the main text (S1 Table). (TIFF) [file pcbi.1006787.s015.tiff]

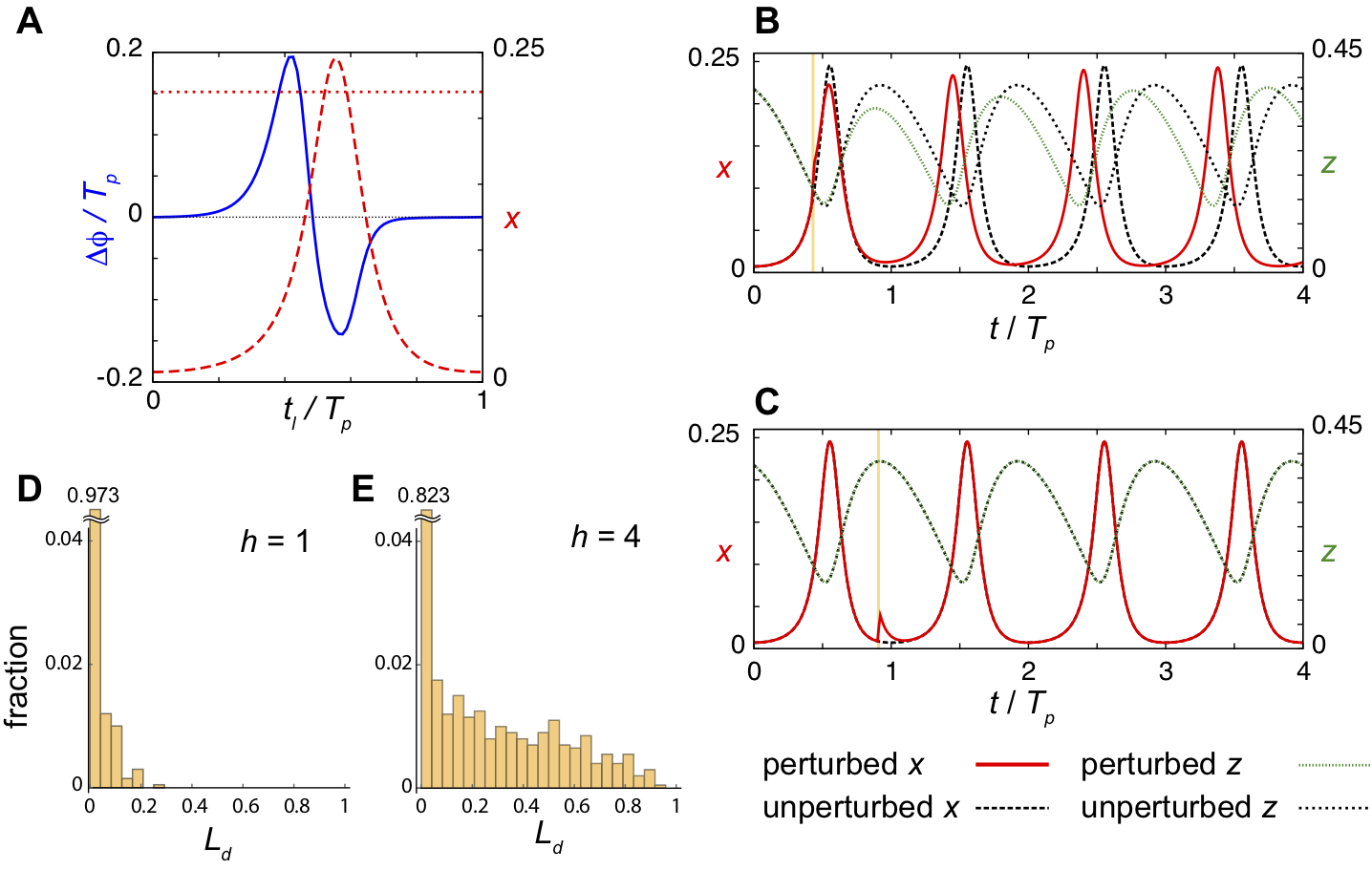

Supplement: S14 Fig — (A) Phase shift Δϕ as a function of the onset of light signals tl with a larger threshold constant Kt in Eq (12) in the main text. The time series of x (red broken line) is also plotted as a reference. The horizontal red dotted line plotted against the right y-axis indicates the value of Kt. (B), (C) Time series of mRNA x and nuclear protein z with light signals administered at (B) tl/Tp = 0.42 and (C) tl/Tp = 0.9 (yellow color). (D), (E) Histograms of the dead zone length Ld for (D) h = 1 and (E) h = 4. Results for 2000 random parameter sets are used. The bin size is 1/24. Parameter values in (A)-(C) are same and listed in S1 Table. Tp = 24, εl = 0.1 and Td = 0.5Tp/24 = 0.5 in (A)-(C). (TIFF) [file pcbi.1006787.s016.tiff]
